# Comprehensive map of visual projection neurons for processing ultraviolet information in the *Drosophila* brain

**DOI:** 10.1002/cne.25068

**Published:** 2020-12-05

**Authors:** Chu‐Yi Tai, An‐Lun Chin, Ann‐Shyn Chiang

**Affiliations:** ^1^ Institute of Biotechnology National Tsing Hua University Hsinchu Taiwan; ^2^ Brain Research Center National Tsing Hua University Hsinchu Taiwan; ^3^ Institute of Systems Neuroscience National Tsing Hua University Hsinchu Taiwan; ^4^ Graduate Institute of Clinical Medical Science China Medical University Taichung Taiwan; ^5^ Institute of Molecular and Genomic Medicine National Health Research Institutes Miaoli County Taiwan; ^6^ Department of Biomedical Science and Environmental Biology Kaohsiung Medical University Kaohsiung Taiwan; ^7^ Kavli Institute for Brain and Mind University of California at San Diego La Jolla California USA

**Keywords:** brain mapping, *Drosophila melanogaster*, spatial navigation, visual pathways

## Abstract

The brain perceives visual information and controls behavior depending on its underlying neural circuits. How UV information is represented and processed in the brain remains poorly understood. In *Drosophila melanogaster*, UV light is detected by the R7 photoreceptor that projects exclusively into the medulla layer 6 (M_6_). Herein, we imaged 28,768 single neurons and identified 238 visual projection neurons linking M_6_ to the central brain. Based on morphology and connectivity, these visual projection neurons were systematically classified into 94 cell types belonging to 12 families. Three tracts connected M_6_ in each optic lobe to the central brain: One dorsal tract linking to the ipsilateral lateral anterior optic tubercle (L‐AOTU) and two medial tracts linking to the ipsilateral ventral medial protocerebrum (VMP) and the contralateral VMP. The M_6_ information was primarily represented in the L‐AOTU. Each L‐AOTU consisted of four columns that each contained three glomeruli. Each L‐AOTU glomerulus received inputs from M_6_ subdomains and gave outputs to a glomerulus within the ellipsoid body dendritic region, suggesting specific processing of spatial information through the dorsal pathway. Furthermore, the middle columns of the L‐AOTUs of both hemispheres were connected via the intertubercle tract, suggesting information integration between the two eyes. In contrast, an ascending neuron linked each VMP to all glomeruli in the bulb and the L‐AOTU, bilaterally, suggesting general processing of information through the ventral pathway. Altogether, these diverse morphologies of the visual projection neurons suggested multi‐dimensional processing of UV information through parallel and bilateral circuits in the *Drosophila* brain.

## Graphical abstract

Insects use UV light as navigation signals to locate the position of the sun. In this study, we construct a comprehensive map of the downstream circuitry of UV‐sensing R7 neurons by analyzing large‐scale 3D neural data at the single‐neuron resolution in *Drosophila*. We find six families of visual projection neurons that connect the optic lobe to the central brain. Our imaging study reveals the morphological diversity of these visual projection neurons of the medulla layer 6 (M_6_) downstream circuitry suggests the multi‐dimensional processing of UV information via hierarchical, multiple‐layer, parallel, and bilateral circuits in the brain. Furthermore, we describe a detailed neuroanatomical description of the lateral anterior optic tubercle (L‐AOTU) and the bulb (BU).

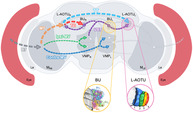

**Abbreviations: Neuropil names**
EB: **E**llipsoid **B**ody
L‐AOTU: **L**ateral **A**nterior **O**ptic **Tu**bercle
BU: **B**ul**b**
M_6_: **M**edulla layer **6**
VMP: **V**entral **M**edial **P**rotocerebrum
Neuron name (The arrow indicates the direction of information flow. The colons indicate a nondirectional connection): MT: **M**_6_ → L‐AO**T**U
MV: **M**_6_ → **V**MP
R7: Retina → M_6_
TB: L‐AO**T**U → **B**U
TT: L‐AO**T**U → L‐AO**T**U
VBT: **V**MP → **B**U::L‐AO**T**U

## 1 INTRODUCTION

Animal navigation relies on vision to generate a spatial map of the external world in the brain. Insects use UV light as navigation signals to locate the position of the sun (el Jundi et al., 2014; Pfeiffer & Homberg, 2007; Warren et al., 2019). In *Drosophila*, the ellipsoid body (EB), a ring‐shaped structure located at the center of the brain, acts as an internal compass for navigation. The neural activity in EB neurons reflects head orientation during locomotion (Fisher et al., 2019; Kim et al., 2019). However, how UV information is represented in the brain and relayed to the EB remains poorly understood.

The *Drosophila* compound eye is composed of about 750 ommatidia, each containing eight photoreceptors: six outer photoreceptors (R1–R6) and two inner photoreceptors (R7 and R8). The R1–R6 photoreceptors express broadband rhodopsin (Rh1) for motion vision, while the R7 and R8 photoreceptors express different narrowband rhodopsin (Rh3–Rh6) for color vision. Visual information from the eye's photoreceptors is conveyed to the optic lobe, which consists of four neuropils: the lamina, medulla, lobula, and lobula plate. While the lamina receives visual input from the R1–R6 photoreceptors, the medulla, which comprises 10 layers (M1–M10), receives visual input from UV‐sensitive R7 photoreceptors and blue‐green‐sensitive R8 photoreceptors (Behnia & Desplan, 2015; Song & Lee, 2018). Color vision extracts spectral information through synapse interactions between R7 and R8 neurons via reciprocal inhibition (Schnaitmann et al., 2018).

The axons of UV‐sensitive R7 neurons terminate exclusively within the layer 6 of the medulla (M_6_). Serial‐section electron microscopy revealed that amacrine Dm8 neurons with dendritic arbors in the M_6_ receive direct synaptic inputs from R7 neurons and connect with transmedulla Tm5c neurons at the lobula. Consistently, functional studies showed that information flows via R7 → Dm8 → Tm5c are necessary for the UV preference behavior in *Drosophila* (Gao et al., 2008; Takemura et al., 2013; Karuppudurai et al., 2014). Conversely, anatomical and functional studies have revealed that other M_6_ output neurons known as MT (also, MC61 or MeTu) terminate at the lateral anterior optical tubercle (L‐AOTU) are necessary for the phototaxis toward UV light (Otsuna et al., 2014). These results suggested that UV information is diverged from M_6_ by different downstream circuits related to control‐specific aspects of behavior. However, the M_6_ output circuits for processing UV information in the *Drosophila* brain remain to be fully determined.

In this study, we performed a large‐scale 3D imaging of single neurons connecting the UV‐receiving M_6_ to the central brain in *Drosophila*. Our results on the topographical organization of 238 reconstructed M_6_ downstream neurons suggested that UV inputs are differentially and hierarchically represented in the brain. Furthermore, the UV information received by the two eyes is integrated and processed in a multilayer manner.

## 2 MATERIALS AND METHODS

### 2.1 Fly stocks

Fly strains (*Drosophila melanogaster*) were reared on a cornmeal‐yeast‐agar medium at 25°C with 70% relative humidity under a 12 h/12 h light/dark cycle. Wild type Canton‐S flies were used for the generation of the representative model of anterior optic tubercle. The GR‐Gal4 and GR‐LexA lines were ordered from the Bloomington *Drosophila* Stock Center and the VT‐Gal4 lines from the Vienna *Drosophila* Resource Center.

### 2.2 Immunohistochemistry

Brain samples were dissected within cold isotonic phosphate‐buffered saline (PBS) and fixed immediately in 4% paraformaldehyde in PBS on ice with microwave irradiation (700 W, 90 s, three times). The samples were fixed again in 4% paraformaldehyde in PBS with 0.25% Triton X‐100 for another session of microwave irradiation (700 W, 90 s, three times). Moreover, the fixed samples were degassed by vacuum in PBS containing 2% Triton X‐100 and 10% normal goat serum (NGS) for 1 h to expel air from the tracheal system and then blocked; then the brain samples were penetrated in the PBS containing 2% Triton X‐100 and 10% NGS at 4°C overnight. For immunohistochemistry, brain samples were incubated in PBS containing 0.25% Triton X‐100 and 1% NGS with mouse anti‐discs large (DLG) antibodies (1:50, antibody 4F3; Developmental Studies Hybridoma Bank [DSHB], University of Iowa, IW) for 2 days at 4°C; biotinylated goat anti‐mouse IgG antibodies (1:250, Molecular Probes, Eugene, OR) overnight at room temperature; and Alexa Fluor 635 streptavidin (Molecular Probes, Eugene, OR) overnight at room temperature. Brain samples were extensively washed in PBS containing 1% Triton X‐100 and 3% sodium chloride at room temperature for 20 min three times between each step. Finally, the immunolabeled brains were cleared and mounted in the *FocusClear*™ (CelExplorer, Taiwan) for confocal imaging.

### 2.3 Confocal imaging and analysis

The morphology of single neurons and expression patterns of driver lines were imaged under a C‐Apochromat 40x/N.A 1.2 water immersion objective lens (scanning speed, 7; line average, 4 times; zoom, 0.7; optical slices, 1 μm; resolution, 1024 × 1024 pixels) on a Zeiss LSM710 confocal microscope. The high‐resolution L‐AOTU and BU models were imaged under a Plan‐Apochromat 63x/N.A 1.4 oil immersion objective lens (scanning speed, 6; line average, 8 times; zoom, 0.7; optical slices, 0.5 μm; resolution, 1024 × 1024 pixels). All individual single‐neuron images were labeled with the mosaic analysis with a repressible cell marker (MARCM) system (Lee & Luo, 1999) and segmented neurons were warped into the *FlyCircuit* standard brain (*FlyCircuit* database, version 1.2; http://www.flycircuit.tw; Chiang et al., 2011; Shih et al., 2015) with Avizo 9.4.0 (Thermo Fisher Scientific, Waltham, MA), as previously described (Chiang et al., 2011; Shih et al., 2015).

## 3 RESULTS

### 3.1 Neural tracts that convey M_6_ visual information to the central brain

**FIGURE 1:**
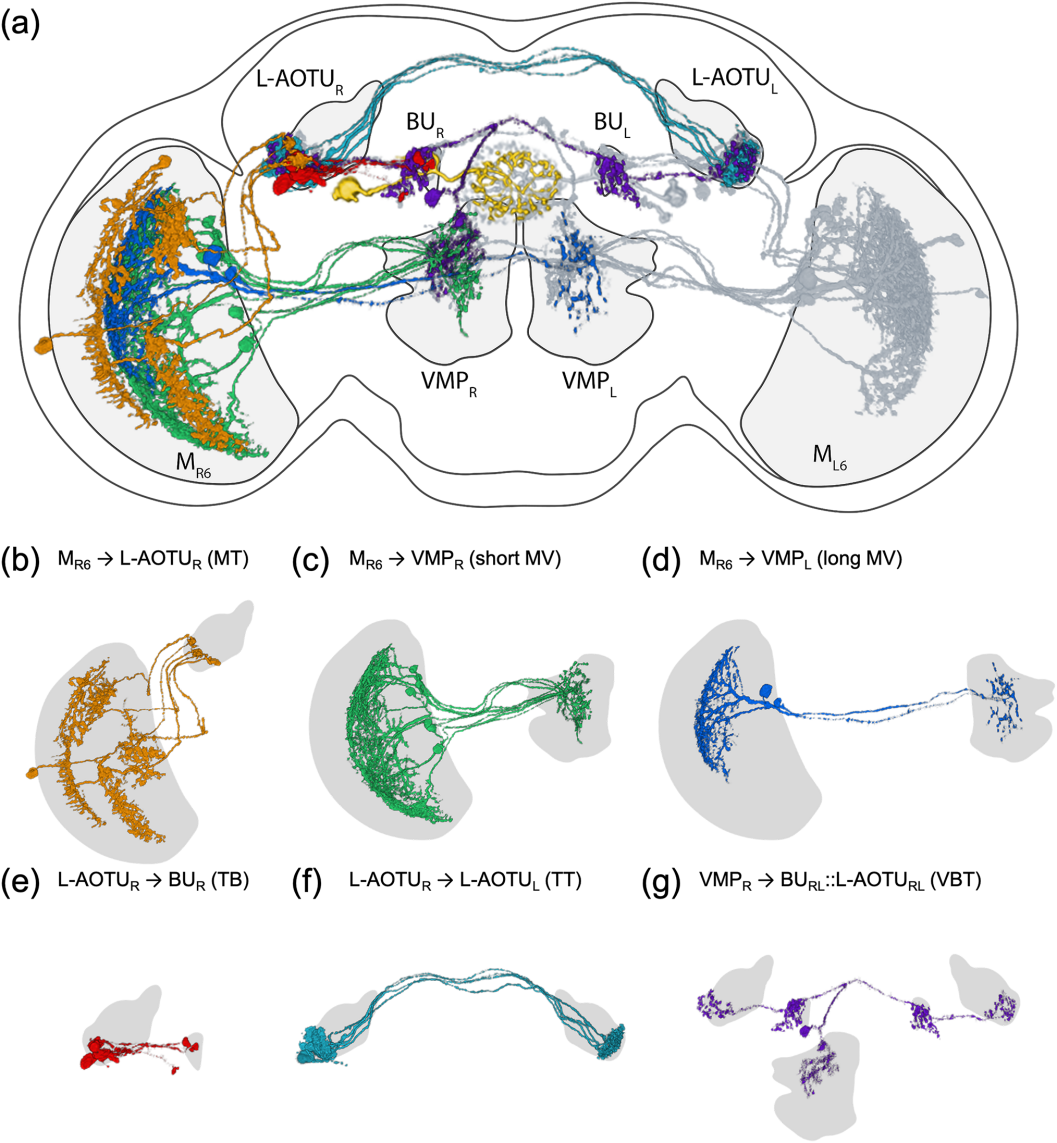
Neural tracts that convey medulla layer 6 (M_6_) visual information to the central brain. (a) Map of the medulla layer 6 (M_6_) downstream circuitry. (b) M_6_ output neurons terminate in the ipsilateral lateral anterior optic tubercle (L‐AOTU), named the MT tract (M_R6_ → L‐AOTU_R_). (c) M_6_ output neurons terminate in the ipsilateral ventral medial protocerebrum (VMP), named the short MV tract (M_R6_ → VMP_R_). (d) M_6_ output neurons terminate in the contralateral VMP, named the long MV tract (M_R6_ → VMP_L_). (e) L‐AOTU output neurons terminate in the ipsilateral bulb (BU), named the TL tract (L‐AOTU_R_ → BU_R_). (f) L‐AOTU output neurons terminate in the contralateral L‐AOTU, named the TT tract (L‐AOTU_R_ → L‐AOTU_L_). (g) A VMP output neuron terminates bilaterally in both the BU and the L‐AOTU, named the VBT tract (VMP_R_ → BU_RL_::L‐AOTU_RL_). An ellipsoid body (EB) ring neuron is shown in yellow. For clarity, all downstream neurons of the M_L6_ are shown in gray [Color figure can be viewed at wileyonlinelibrary.com]

UV‐sensing R7 neurons send axonal terminals to M_6_. Through the analysis of spatial connectivity between the neuropils of 28,608 single neurons collected in the *FlyCircuit* database, we constructed a comprehensive map of the downstream circuitry of R7 neurons (Figure 1a). The downstream neurons of the M_R6_ were classified into six families, based on their neuropil connectivity differences (Figure 1b–g). Three neuronal families diverged from the M_6_: one family terminated in the ipsilateral L‐AOTU via the dorsal tract (named the MT tract, Figure 1b); another family terminated in the ipsilateral VMP via the short medial tract (named the short MV tract, Figure 1c); finally, another family terminated in the contralateral VMP via the long medial tract (named the long MV tract, Figure 1d). Two other neuronal families diverged from the L‐AOTU: one terminated in the ipsilateral BU (named the TB tract, Figure 1e) while the other in the contralateral L‐AOTU (named the TT tract, Figure 1f). The remaining neuronal family branched from the VMP and bilaterally terminated to the BU and the L‐AOTU of both brain hemispheres (named the VBT tract, Figure 1g). Thus, UV information at M_R6_ was relayed to three pairs of neuropils (i.e., L‐AOTU_RL_, VMP_RL_, and BU_RL_) by six neuronal families. Meanwhile, M_L6_ downstream neurons exhibited mirror circuitry of M_R6_ downstream neurons. Altogether, these results suggested that each of these six neuropils receives UV information from both eyes.

**FIGURE 2:**
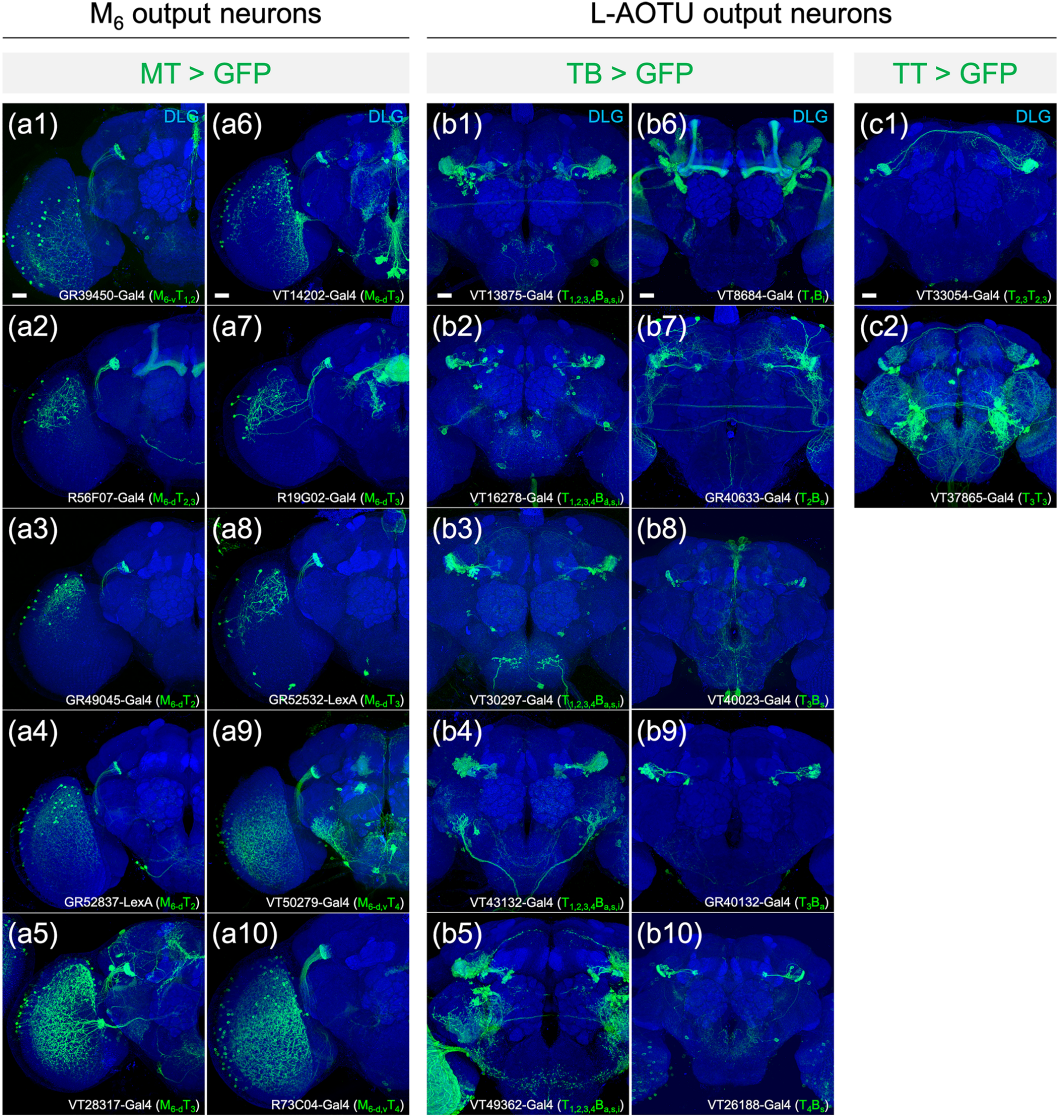
Expression patterns of Gal4 and LexA drivers in MT, TB, and TT neurons. (a1–a10) Expression of medulla layer 6 (M_6_) output MT neuron drivers. (b1–b10) Expression of lateral anterior optic tubercle (L‐AOTU) output TB neuron drivers. (c1–c2) Expression of L‐AOTU output TT neurons drivers. GFP expression patterns (green) and names of Gal4 and LexA drivers (bottom). Brain neuropils were immunostained with anti‐DLG antibodies (blue). Images are frontal views of confocal projections of several adjacent optical sections unless otherwise specified. Scale bar: 20 μm [Color figure can be viewed at wileyonlinelibrary.com]

**FIGURE 3:**
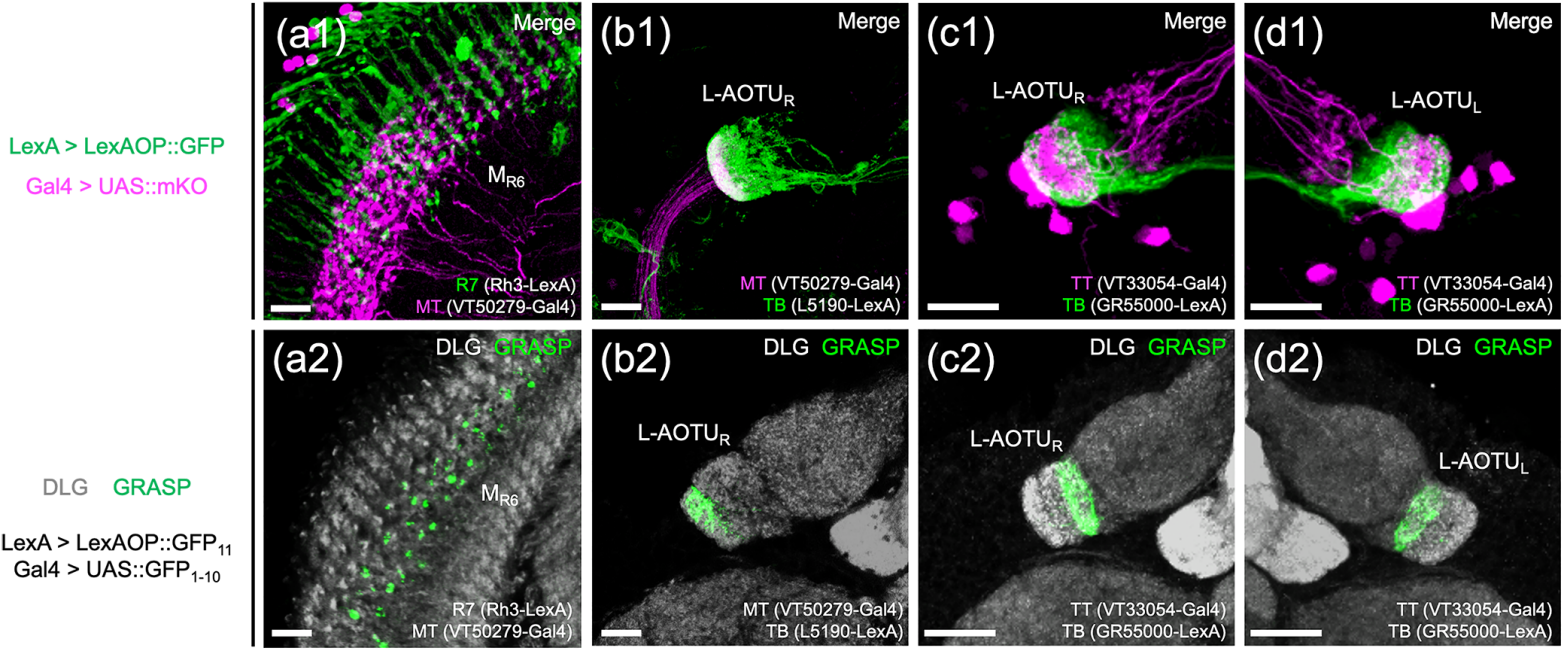
Anatomical connection from UV‐sensing R7 photoreceptors to the central brain. (a) Connection between R7 and the MT. Overlap of double labeling between R7 (labeled with Rh3‐LexA, green) and the MT (labeled with VT50279‐Gal4, magenta) in the medulla layer 6 (M_6_) (a1). The green fluorescent protein reconstitution across synaptic partners (GRASP) signals between R7 and the MT show in the M_6_ (a2). (b) Connection between the MT and the TB. Overlap of double labeling between the MT (labeled with VT50279‐Gal4, magenta) and the TB (labeled with L5190‐LexA, green) in the lateral anterior optic tubercle (L‐AOTU) (b1). The GRASP signals between the MT and the TB show in the L‐AOTU (b2). (c–d) Connection between the TB and the TT of the two hemispheres. Overlap of double labeling between the TB (labeled with GR55000‐LexA, green) and the TT (labeled with VT33054‐Gal4, magenta) in the L‐AOTU_R_ (c1) and the L‐AOTU_L_ (d1). The GRASP signals between the TB and the TT show in the L‐AOTU_R_ (c2) and the L‐AOTU_L_ (d2). Brain neuropils were immunostained with anti‐DLG antibodies (gray). Images are frontal views of confocal projections of several adjacent optical sections unless otherwise specified. Scale bar: 20 μm [Color figure can be viewed at wileyonlinelibrary.com]

Upon examining the expression patterns of more than 5000 Gal4 and LexA drivers, we identified 22 with a specific expression in MT, TB, or TT neurons (Figure 2 and Table 1). No driver showed specific expression in short MV, long MV, or VBT neurons. To test neuronal connectivity between R7 neurons, MT, TB, and TT neurons, we used the two‐color labeling with selected Gal4 drivers expressed in red fluorescent proteins (mKO) and LexA drivers expressed in green fluorescent proteins (GFP). Two‐color labeling demonstrated the physical connections that M_6_ input R7 neurons overlapped with M_6_ output MT neurons in the M_6_ (Figure 3a1), L‐AOTU input MT neurons overlapped with L‐AOTU output TB neurons in the L‐AOTU (Figure 3b1), and L‐AOTU commissural TT neurons overlapped with L‐AOTU output TB neurons in the two L‐AOTUs (Figure 3c1, d1). To further confirm synaptic connectivity between pre‐ and post‐synaptic neurons, we used the method of green fluorescent protein reconstitution across synaptic partners (GRASP) with selected Gal4 and LexA drivers respectively expressed two split‐GFP fragments—spGFP_1‐10_ and spGFP_11_ (Feinberg et al., 2008). GRASP signal was observed in the synaptic connections between M_6_ input R7 neurons and M_6_ output MT neurons (Figure 3a2), L‐AOTU input MT neurons and L‐AOTU output TB neurons (Figure 3b2), and L‐AOTU commissural TT neurons and L‐AOTU output TB neurons (Figure 3c2,d2). Consequently, these findings suggested that UV information is conveyed from the medulla of the optic lobe to the EB of the central brain via two UV pathways, namely, R7 → M_6_ → ipsilateral L‐AOTU → ipsilateral BU and R7 → M_6_ → ipsilateral L‐AOTU → contralateral L‐AOTU → contralateral BU.

**TABLE 1:**
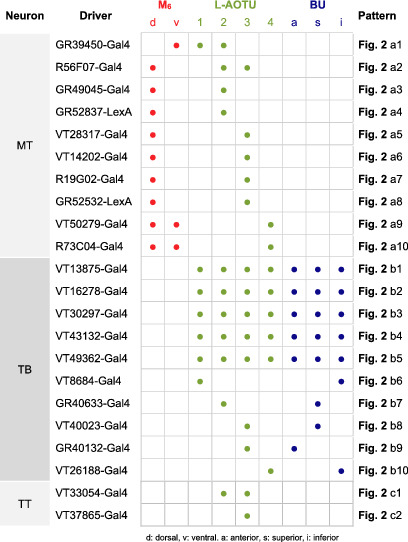
Characterization of the innervation pattern of 22 drivers expressed in MT, TB, and TT neurons [Color table can be viewed at wileyonlinelibrary.com]

Abbreviations: a, anterior; d, dorsal; i, inferior; s, superior; v, ventral.

### 3.2 The L‐AOTU consists of four columns

**FIGURE 4:**
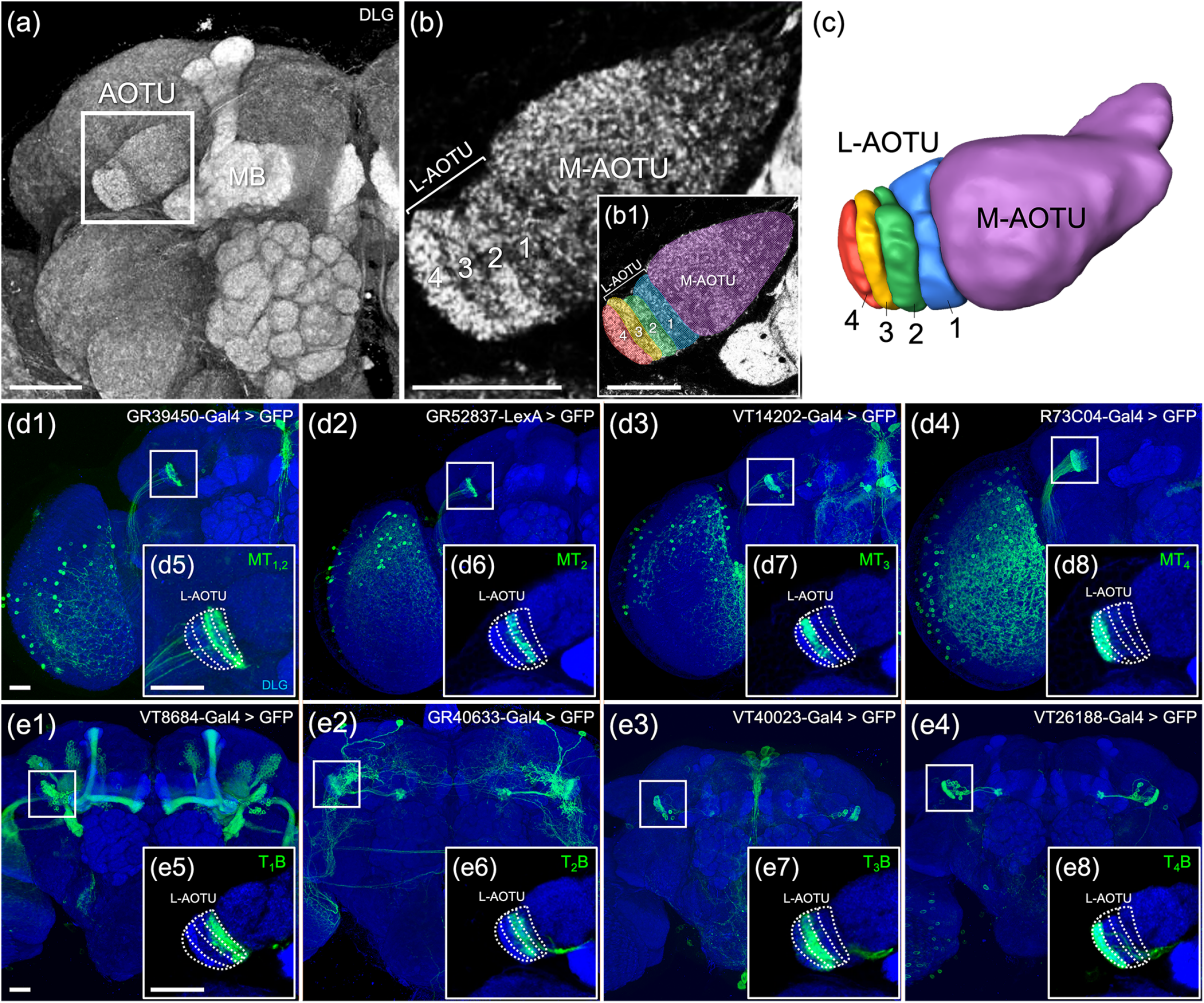
The lateral anterior optic tubercle (L‐AOTU) consists of four columns. (a) The anterior optic tubercle (AOTU) is located near the frontal surface of the brain at the side of the mushroom (MB). (b) The AOTU consists of the medial AOTU (M‐AOTU) and the lateral AOTU (L‐AOTU) and locates near the frontal surface of the brain. Imaging of anti‐discs large (DLG) stained neuropils (gray) reveals clear structural boundaries, including the vertical strata of the L‐AOTU. (b1) Each color reveals a demarcated substructure in the L‐AOTU. (c) Volume models of four columns in the L‐AOTU. (d1–d4) Expression of column‐specific Gal4 and LexA drivers in input MT neurons that were visualized with mCD8::GFP. (d5–d8) High‐magnification image in the L‐AOTU of column‐specific input MT neurons (boxed inset). (e1–e4) Expression of column‐specific Gal4 and LexA drivers expressed in output TB neurons that were visualized with mCD8::GFP. (e5–e8) High‐magnification image in the L‐AOTU of column‐specific output TB neurons (boxed inset). Brain structures immunostained with anti‐DLG antibodies (gray in a and b; blue in d and e). Images are frontal views of confocal projections of several adjacent optical sections unless otherwise specified. Scale bars: 20 μm [Color figure can be viewed at wileyonlinelibrary.com]

**FIGURE 5:**
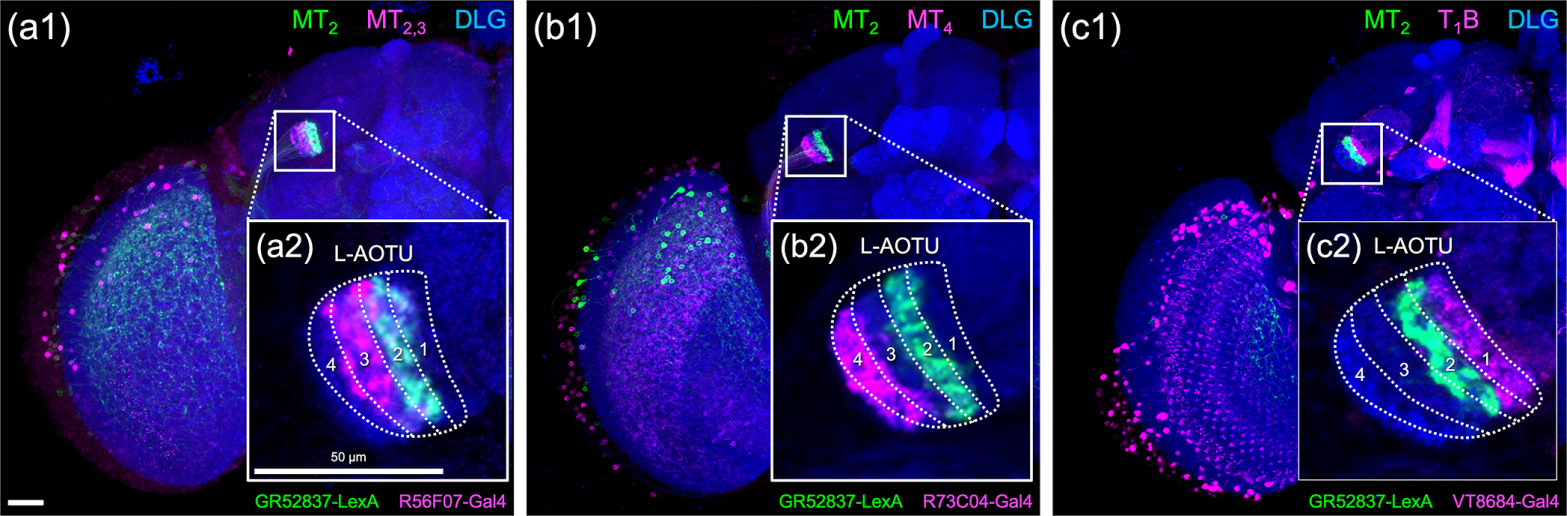
Double labeling revealed the lateral anterior optic tubercle (L‐AOTU) contains four columns. Double labeling of column‐specific MT and TB drivers. (a) The axonal innervation of MT_2_ is in L‐AOTU_2_ (labeled with GR52837‐LexA, green) and the axonal innervation of MT_2,3_ is in L‐AOTU_2,3_ (labeled with R56F07‐Gal4, magenta), confirming previous findings that the intermediate lateral L‐AOTU is L‐AOTU_2_ and L‐AOTU_3_. (b) The axonal innervation of MT_2_ is in L‐AOTU_2_ (labeled with GR52837‐LexA, green) and the axonal innervation of MT_4_ is in L‐AOTU_4_ (labeled with R73C04‐Gal4, magenta), confirming previous findings that the lateral L‐AOTU is L‐AOTU_4_. (c) The axonal innervation of MT_2_ is in L‐AOTU_2_ (labeled with GR52837‐LexA, green) and the dendritic innervation of T_1_B is in L‐AOTU_1_ (labeled with VT8684‐Gal4, magenta). (a2–c2) High‐magnification image of the L‐AOTU (boxed inset). Brain structures immunostained with anti‐DLG antibodies (blue). Images are frontal views of confocal projections of several adjacent optical sections unless otherwise specified. Scale bars: 20 μm (a1–c1) and 50 μm (a2–c2) [Color figure can be viewed at wileyonlinelibrary.com]

The L‐AOTU is located at the dorsal–frontal surface, between the mushroom body lobes and the lateral horn in each hemisphere (Figure 4a). Previous studies defined three columns in L‐AOTU: the medial L‐AOTU, the intermediate lateral L‐AOTU, and the lateral L‐AOTU (Omoto et al., 2017; Timaeus et al., 2020). Here, we performed immunolabeling with anti‐DLG antibodies using high‐resolution confocal microscopy revealed that the L‐AOTU is subdivided into four vertical columns by the aggregation of DLG synaptic proteins (Figure 4b). The obvious boundary allowed us to manually demarcate individual L‐AOTU columns (L‐AOTU_1_, L‐AOTU_2_, L‐AOTU_3_, and L‐AOTU_4_) from medial to lateral (Figure 4b1), and to visualize their spatial relationships (Figure 4c). Furthermore, each L‐AOTU column expressed Gal4 or LexA drivers in the column‐specific input MT tract (Figure 4d1–d4) and the column‐specific output TB tract (Figure 4e1–e4). Double labeling with two L‐AOTU column‐specific drivers showed the four L‐AOTU columns in each brain hemisphere in the same fly (Figure 5). Indeed, we found that the R56F07‐Gal4 driver, which previously defined as located in the intermediate lateral L‐AOTU (Omoto et al., 2017), contains two columns, namely, the L‐AOTU_2_ and the L‐AOTU_3_ (Figure 5a and Table 2). Intriguingly, only the L‐AOTU_2_ and the L‐AOTU_3_ in both hemispheres were linked by the commissural TT tract formed by the L‐AOTU_R2,R3_ → L‐AOTU_L2,L3_; L‐AOTU_L2,L3_ → L‐AOTU_R2,R3_; L‐AOTU_R3_ → L‐AOTU_L3_; and L‐AOTU_L3_ → L‐AOTU_R3_ neurons (Figure 2c1–c2, and Table 1).

**TABLE 2:** Comparison of lateral anterior optic tubercle (L‐AOTU) subdivisions from the current study and previous studies

Current study (2020)		Omoto et al. (2017)		Timaeus et al. (2020)	
**1**	VT8684‐Gal4	Intermediate (**im**)	R25C04‐Gal4	Medial (**m**)	R20B05‐LexA
**2**	GR40683‐Gal4	Intermediate lateral (**il**)	R56F07‐Gal4	Central (**c**)	Anti‐connectin
**3**	VT40023‐Gal4				
**4**	R73C04‐Gal4	Lateral (**l**)	R73C04‐Gal4	Lateral (**l**)	R85F05‐Gal4

### 3.3 Cell typing and nomenclature

**FIGURE 6:**
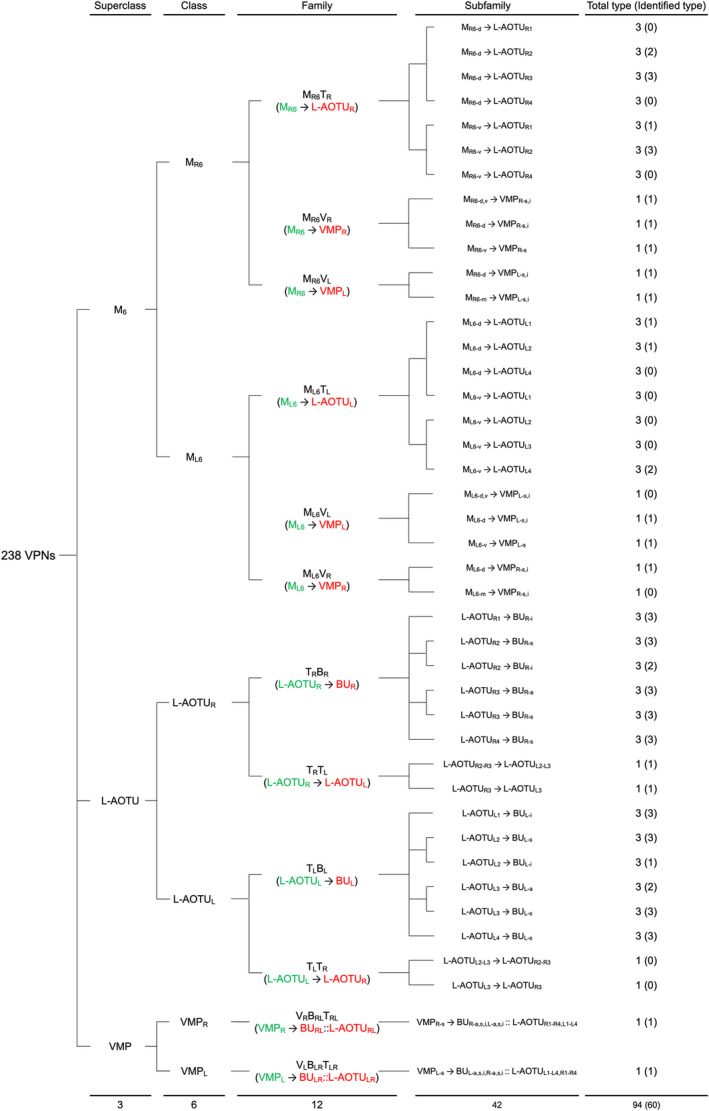
Classifying single visual projection neurons (VPNs) into 94 cell types (including 60 identified types). Classification tree diagram of 238 single visual projection neurons (VPNs). The arrow indicates the direction of the information flow. The regions where dendrites are located are shown in green and axons in red. The numbers in brackets indicate the types identified from our dataset of single neurons. M_6_, medulla layer 6; L‐AOTU, lateral anterior optic tubercle; VMP, ventral medial protocerebrum; BU, bulb; d, dorsal; m, medial; v, ventral; a, anterior; s, superior; I, inferior. See also Tables 3 and 4 [Color figure can be viewed at wileyonlinelibrary.com]

We imaged the 160 single neurons that were stochastically labeled by MARCM (Lee & Luo, 1999) using specific Gal4 drivers expressed in M_6_ downstream neurons (Figure 2). Together with 78 related neurons in the *FlyCircuit* database, we classified and named a total of 238 R7 downstream neurons (Table 3) into 94 cell types (including predicted types), belonging to 42 subfamilies, 12 families, 6 classes, and 3 superclasses (Figure 6 and Table 3). Neurons of the same cell type were morphologically indistinguishable, connected the same neuropils, and had a similar spatial distribution for both dendritic and axonal terminals. Neurons with dendrites and axons that innervated the same neuropils belonged to the same family. Within the same family, neurons with axonal terminals distributed in the same neuropil subdivision belonged to the same subfamily. Neurons with dendrites that innervated the same neuropil belonged to the same class. Neurons with mirror morphology between the two hemispheres belonged to the same superclass.

**TABLE 3:**
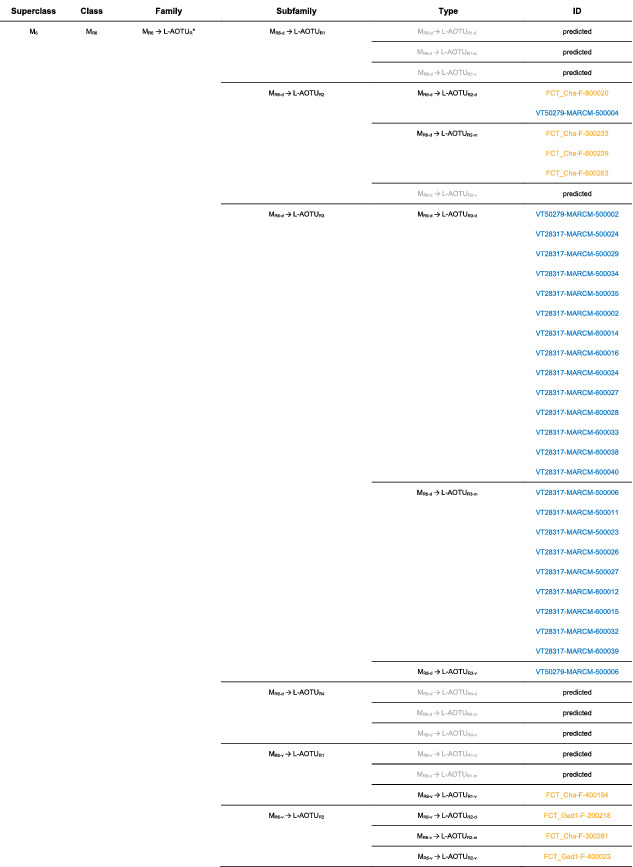
Neurons are identified from *Flycircuit* (orange), selected Gal4 (blue), and predicted type (gray) [Color table can be viewed at wileyonlinelibrary.com]

*Note*: *The corresponding name previously used in Drosophila.

**The corresponding name previously used in Drosophila.

For the systematic analysis of 238 single neurons, each cell type was assigned a simple name based on their dendritic and axonal distributions. For example, M_R6‐d_ → L‐AOTU_R2‐d_ (named M_R6‐d_T_R2‐d_, Table 4, and Table 5) represents the MT neuron with dendritic arbors in the dorsal region of the right M_6_ and the axon terminals in the dorsal glomerulus of the right L‐AOTU_2_. Upon examining thousands of single‐neuron images and driver expressions, we found that these visual projection neurons (VPNs) were highly stereotyped and left–right symmetric in all cases from different individual neurons. This allowed us to predict additional 34 types of M_6_ downstream neurons (i.e., M_L6‐v_ → L‐AOTU_L4‐v_ and M_R6‐v_ → L‐AOTU_R4‐v_ were predicted from the specific MT_4_ drivers, while and M_L6‐d,v_ → VMP_L‐s,i_ was predicted from the symmetrical neuronal partner, M_R6‐d,v_ → VMP_R‐s,i_). Using this systematic nomenclature system, we provided a detailed list of all identified and predicted cell types of the M_6_ downstream neurons (See Tables 3 and 4 for VPN nomenclature and synonyms).

**TABLE 4:**
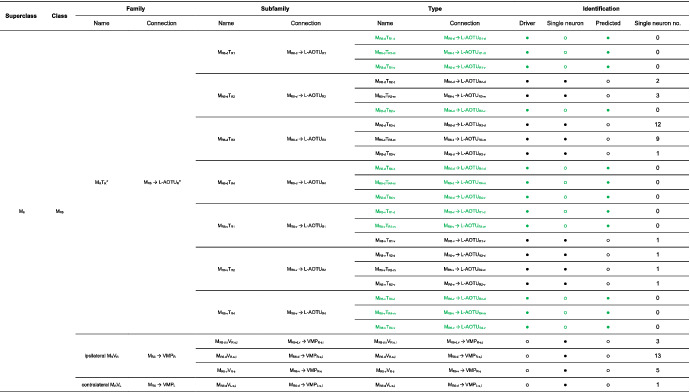
Identified cell types (black) and predicted type from specific drivers (green) and symmetrical neuronal partner (red)

**TABLE 5:** Lookup table of neuropil abbreviations

M_6_	Medulla, layer 6	L‐AOTU_L1‐m_	Left L‐AOTU, 1st column, medial glomerulus
**M**_**R6**_	Right M_6_	**L‐AOTU**_**L1‐v**_	Left L‐AOTU, 1st column, ventral glomerulus
**M**_**R6‐d**_	Right M_6_, dorsal part	**L‐AOTU**_**L2**_	Left L‐AOTU, 2nd column
**M**_**R6‐m**_	Right M_6_, medial part	**L‐AOTU**_**L2‐d**_	Left L‐AOTU, 2nd column, dorsal glomerulus
**M**_**R6‐v**_	Right M_6_, ventral part	**L‐AOTU**_**L2‐m**_	Left L‐AOTU, 2nd column, medial glomerulus
**M**_**L6**_	Left M_6_	**L‐AOTU**_**L2‐v**_	Left L‐AOTU, 2nd column, ventral glomerulus
**M**_**L6‐d**_	Left M_6_, dorsal part	**L‐AOTU**_**L3**_	Left L‐AOTU, 3rd column
**M**_**L6‐m**_	Left M_6_, medial part	**L‐AOTU**_**L3‐d**_	Left L‐AOTU, 3rd column, dorsal glomerulus
**M**_**L6‐v**_	Left M_6_, ventral part	**L‐AOTU**_**L3‐m**_	Left L‐AOTU, 3rd column, medial glomerulus
**L‐AOTU**	Lateral Anterior Optic Tubercle	**L‐AOTU**_**L3‐v**_	Left L‐AOTU, 3rd column, ventral glomerulus
**L‐AOTU**_**R**_	Right L‐AOTU	**L‐AOTU**_**L4**_	Left L‐AOTU, 4th column
**L‐AOTU**_**R1**_	Right L‐AOTU, 1st column	**L‐AOTU**_**L4‐d**_	Left L‐AOTU, 4th column, dorsal glomerulus
**L‐AOTU**_**R1‐d**_	Right L‐AOTU, 1st column, dorsal glomerulus	**L‐AOTU**_**L4‐m**_	Left L‐AOTU, 4th column, medial glomerulus
**L‐AOTU**_**R1‐m**_	Right L‐AOTU, 1st column, medial glomerulus	**L‐AOTU**_**L4‐v**_	Left L‐AOTU, 4th column, ventral glomerulus
**L‐AOTU**_**R1‐v**_	Right L‐AOTU, 1st column, ventral glomerulus	**BU**	Bulb
**L‐AOTU**_**R2**_	Right L‐AOTU, 2nd column	**BU**_**R**_	Right BU
**L‐AOTU**_**R2‐d**_	Right L‐AOTU, 2nd column, dorsal glomerulus	**BU**_**R‐a**_	Right BU, anterior group
**L‐AOTU**_**R2‐m**_	Right L‐AOTU, 2nd column, medial glomerulus	**BU**_**R‐s**_	Right BU, superior group
**L‐AOTU**_**R2‐v**_	Right L‐AOTU, 2nd column, ventral glomerulus	**BU**_**R‐i**_	Right BU, inferior group
**L‐AOTU**_**R3**_	Right L‐AOTU, 3rd column	**BU**_**L**_	Left BU
**L‐AOTU**_**R3‐d**_	Right L‐AOTU, 3rd column, dorsal glomerulus	**BU**_**L‐a**_	Left BU, anterior group
**L‐AOTU**_**R3‐m**_	Right L‐AOTU, 3rd column, medial glomerulus	**BU**_**L‐s**_	Left BU, superior group
**L‐AOTU**_**R3‐v**_	Right L‐AOTU, 3rd column, ventral glomerulus	**BU**_**L‐i**_	Left BU, inferior group
**L‐AOTU**_**R4**_	Right L‐AOTU, 4th column	**VMP**	Ventral Medial Protocerebrum
**L‐AOTU**_**R4‐d**_	Right L‐AOTU, 4th column, dorsal glomerulus	**VMP**_**R**_	Right VMP
**L‐AOTU**_**R4‐m**_	Right L‐AOTU, 4th column, medial glomerulus	**VMP**_**R‐s**_	Right VMP, superior part
**L‐AOTU**_**R4‐v**_	Right L‐AOTU, 4th column, ventral glomerulus	**VMP**_**R‐i**_	Right VMP, inferior part
**L‐AOTU**_**L**_	Left L‐AOTU	**VMP**_**L**_	Left VMP
**L‐AOTU**_**L1**_	Left L‐AOTU, 1st column	**VMP**_**L‐s**_	Left VMP, superior part
**L‐AOTU**_**L1‐d**_	Left L‐AOTU, 1st column, dorsal glomerulus	**VMP**_**L‐i**_	Left VMP, inferior part

### 3.4 MT neurons

**FIGURE 7:**
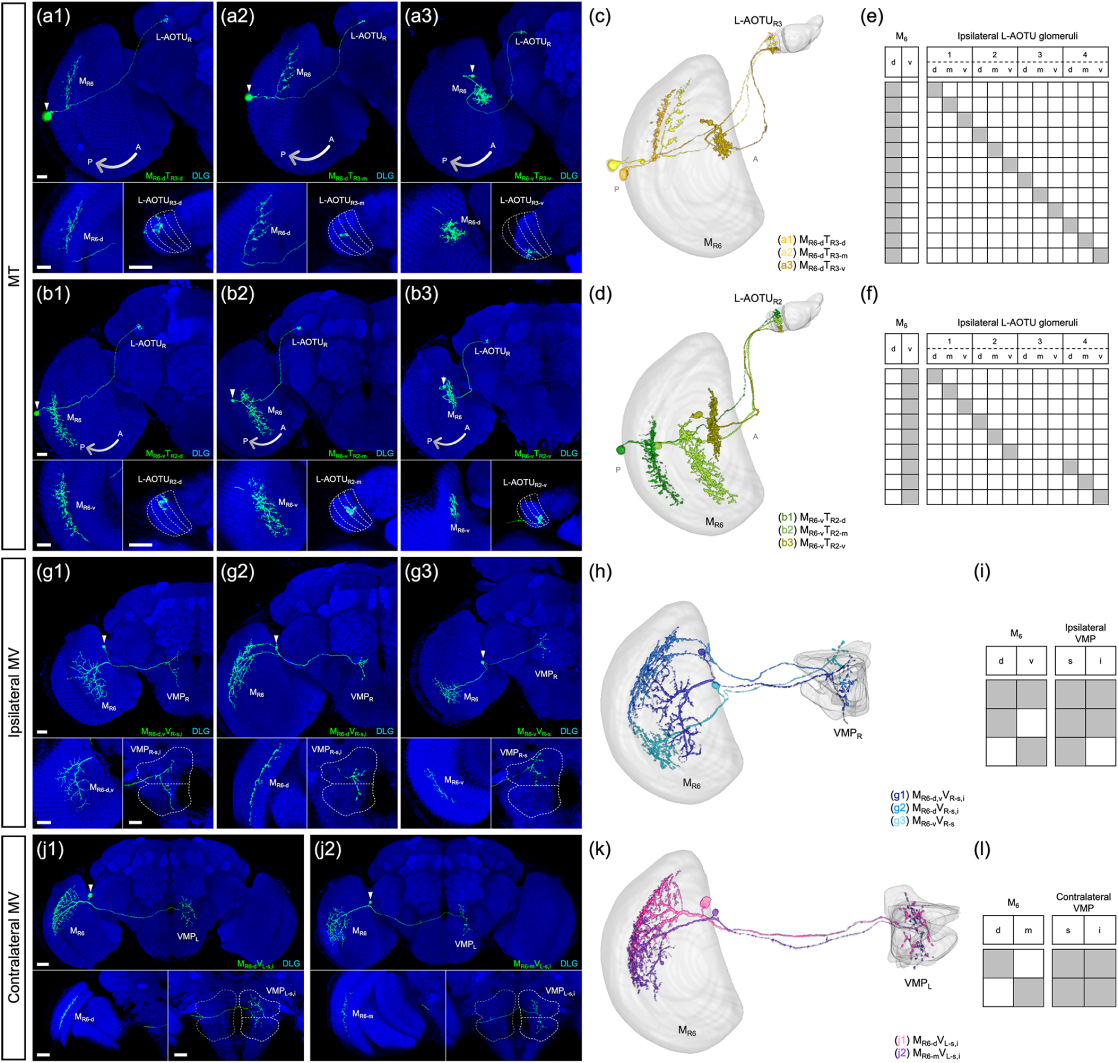
Connectivity analysis for medulla layer 6 (M_6_) output neurons MT, ipsilateral MV, and contralateral MV. (a) Three representative MT neurons of the M_R6‐d_T_R3_ subfamily. (b) Three representative MT neurons of the M_R6‐v_T_R2_ subfamily. Brain structures immunostained with anti‐DLG antibodies (blue). (c–d) Spatial distributions of three representative MT neurons of the same subfamily. Each MT neuron is registered to a standard brain and is shown in a different color. (e–f) Gray indicates the innervation patterns of MT neurons between medulla layer 6 (M_6_) domains and lateral anterior optic tubercle (L‐AOTU) glomeruli. (g) Three representative ipsilateral MV neurons of different subfamilies. Brain structures immunostained with anti‐DLG antibodies (blue). (h) Spatial distribution of three representative ipsilateral MV neurons of different subfamilies. Each ipsilateral MV neuron is registered to a standard brain and shown in a different color. (i) Gray indicates the innervation patterns of MV neurons between M_6_ domains and ipsilateral ventral medial protocerebrum (VMP) domains. (j) Two representative contralateral MV neurons of different subfamilies. Brain structures immunostained with anti‐DLG antibodies (blue). (k) Spatial distribution of two representative contralateral MV neurons of different subfamilies. Each contralateral MV neuron is registered to a standard brain and shown in a different color. (l) Gray indicates the innervation patterns of contralateral MV neurons between M_6_ domains and contralateral VMP domains. Images are frontal views of confocal projections of several adjacent optical sections unless otherwise specified. Scale bars: 20 μm [Color figure can be viewed at wileyonlinelibrary.com]

All MT neurons linked the M_6_ to the ipsilateral L‐AOTU and shared similar morphology (Figure 7a–b). Each MT neuron had small‐field dendritic arbors in the subdomain of the M_6_ and axonal terminals in a single glomerulus of the L‐AOTU. Based on the differences in the connection between the M_6_ subdomains and the L‐AOTU glomeruli, we classified the MT neurons into 21 cell types (including predicted cell types) belonging to seven subfamilies in each hemisphere (Figure 6 and Tables 3 and 4 for systematic analysis and a full list of cell types). The dendrites of MT neurons covered the entire M_6_ from dorsal to ventral and from anterior to posterior, revealing tilling innervation patterns in the M_6_ (Figure 7a–d). The axons of MT neurons further subdivided the L‐AOTU into 12 glomeruli, and each L‐AOTU column contained three glomeruli from dorsal, medial, to ventral (Figure 7c–d). Altogether, MT neurons exhibited the following wiring principles: (a) L‐AOTU_3_ glomeruli receive visual inputs exclusively from dorsal M_6_, but glomeruli in the L‐AOTU_1_, L‐AOTU_2_, and L‐AOTU_4_ receive visual inputs from both the dorsal M_6_ and the ventral M_6_ (Figure 7e–f); (b) each L‐AOTU column receives visual inputs from both the posterior M_6_ and the anterior M_6_ (as shown by specific MT drivers) (Figure 2a); (c) the dorsal glomerulus of each L‐AOTU column receives visual inputs from the posterior M_6_, while the ventral glomerulus of each L‐AOTU column receives visual inputs from the anterior M_6_ (as indicated by a single MT neurons) (Figure 7c–d), consistently with previous reports (Timaeus et al., 2020); and (d) each MT neuron has symmetrical neuronal partners in the contralateral brain hemisphere (i.e., M_R6‐d_ → L‐AOTU_R2‐m_ and M_L6‐d_ → L‐AOTU_L2‐m_) (Figure 11a, yellow line).

### 3.5 MV neurons

The morphological analysis of thousands of single neurons allowed us to identify two families of M_6_ output neurons: one family linking the M_6_ to the ipsilateral VMP (named the ipsilateral MV, Figure 7g), and another family linking the M_6_ to the contralateral VMP (named the contralateral MV, Figure 7j). The VMP is located at the most posterior region of the brain, below the protocerebral bridge of each hemisphere. Each MV neuron had small‐field dendritic arbors in a subdomain of the M_6_ and axon terminals in a subdomain of the VMP. Based on the differences in the connections between the M_6_ subdomains and the VMP subdomains, the ipsilateral MV neurons and the contralateral MV neurons had three subfamilies and two subfamilies, respectively, per brain hemisphere, and each subfamily only had one cell type (including predicted cell types) (Figure 6 and Tables 3 and 4). The morphology of three types of ipsilateral MV neurons showed that two types had axon terminals in the medial region of the superior and inferior VMPs (Figure 7g1–g2), while the remaining one had axon terminals exclusively in the medial regions of superior VMP (Figure 7g3). Furthermore, all contralateral MV neurons had axon terminals in the medial region of the superior and inferior VMPs (Figure 7j1–j2). Consequently, each VMP receives the convergent visual inputs from both ipsilateral M_6_ and contralateral M_6_ (Figure 11a, green and blue line).

### 3.6 TB neurons

**FIGURE 8:**
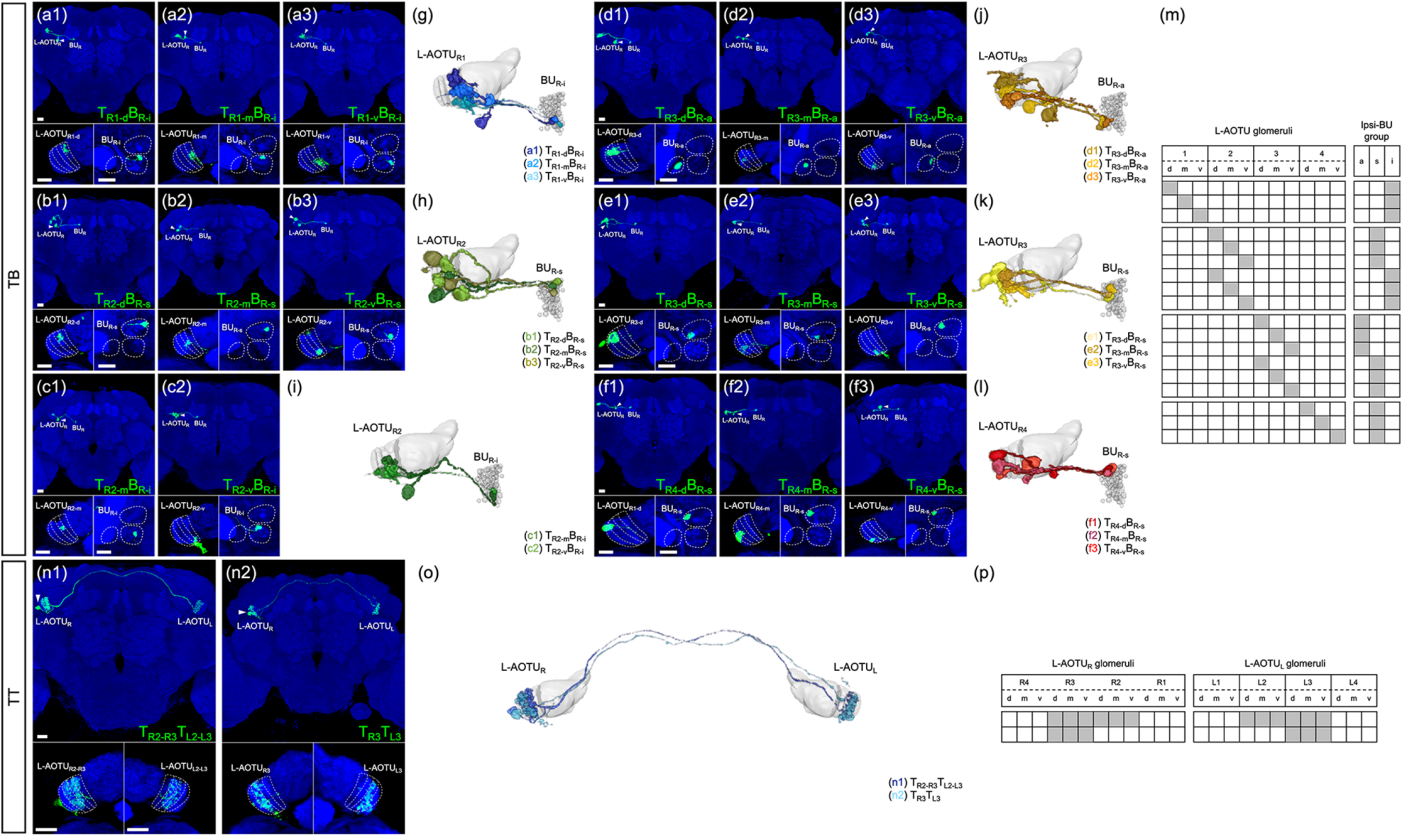
Connectivity analysis for lateral anterior optic tubercle (L‐AOTU) output neurons TB and TT. (a) Three representative TB neurons of the T_R1_B_R‐i_ subfamily. (b) Three representative TB neurons of the T_R2_B_R‐s_ subfamily. (c) Two representative TB neurons of the T_R2_B_R‐i_ subfamily. (d) Three representative TB neurons of the T_R3_B_R‐a_ subfamily. (e) Three representative TB neurons of the T_R3_B_R‐s_ subfamily. (f) Three representative TB neurons of the T_R4_B_R‐s_ subfamily. Brain structures immunostained with anti‐DLG antibodies (blue). (g–l) Spatial distribution of three representative TB neurons of the same subfamily. Each TB neuron is registered to a standard brain and shown in a different color. (m) Gray indicates the innervation patterns of the TB neurons between L‐AOTU glomeruli and ipsilateral BU groups. (n) Two representative TT neurons of different subfamilies. Brain structures immunostained with anti‐DLG antibodies (blue). (o) Spatial distribution of two representative TT neurons of the different subfamilies. Each TT neuron is registered to a standard brain and shown in a different color. (p) Gray indicates the innervation patterns of the TT neurons between the right and left L‐AOTU glomeruli. Images are mostly frontal views of confocal projections of several adjacent optical sections unless otherwise specified. Scale bars: 20 μm [Color figure can be viewed at wileyonlinelibrary.com]

**FIGURE 9:**
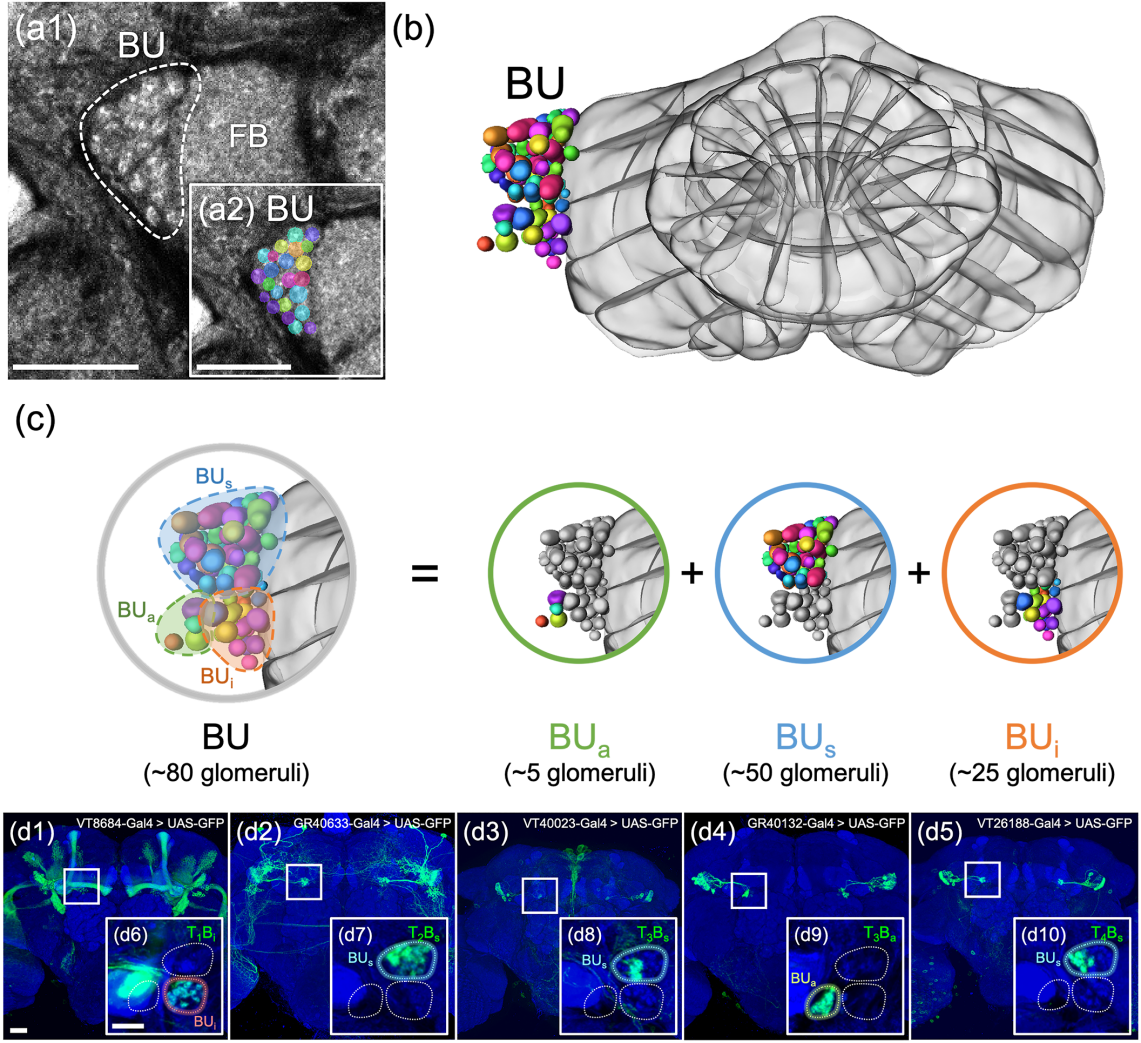
The bulb (BU) consists of ~80 microglomeruli divided into three groups. (a1) The bulb (BU) is located in the central brain at the side of the fan‐shaped body (FB). (a2) Imaging of anti‐discs large (DLG) stained neuropils (gray) reveals clear structural boundaries, including the granular structure of BU. Each color reveals a demarcated substructure in the BU. (b) Volume models of 80 microglomeruli in the BU. (c) Volume models of BU groups: The anterior group (BU_a_), the superior group (BU_s_), and the inferior (BU_i_) group. (d1–d5) Expression of column‐specific Gal4 drivers in TB that were visualized with mCD8::GFP. (e5–e8) High‐magnification image in the BU groups of TB neurons (boxed inset). Brain structures immunostained with anti‐DLG antibodies (blue). Images are mostly frontal views of confocal projections of several adjacent optical sections unless otherwise specified. a, anterior; s, superior; i, inferior. Scale bars: 20 μm [Color figure can be viewed at wileyonlinelibrary.com]

All TB neurons linked the L‐AOTU to the ipsilateral BU (Figure 8a–f). Each TB neuron had dendritic arbors in a single glomerulus of the L‐AOTU and axon terminals in a single glomerulus of BU. Immunolabeling of synaptic proteins with anti‐DLG antibodies revealed that each BU consisted of approximately 80 microglomeruli (Figure 9), which were divided into three groups: ~5 anterior microglomeruli (BU_a_), 50 superior microglomeruli (BU_s_) and 25 inferior microglomeruli (BU_i_) (Figure 9c). Based on the differences in the connections between the 12 glomeruli of the L‐AOTU and the three groups of BU microglomeruli, we classified TB neurons into 18 cell types (including predicted cell types) belonging to six subfamilies in each hemisphere (Figure 6 and Tables 3 and 4 for systematic analysis and a full list of cell types). The dendrites of TB neurons covered the entire L‐AOTU glomeruli from the L‐AOTU_1_ to the L‐AOTU_4_, revealing that dendrites of different types of TB neurons overlap in the L‐AOTU. Interestingly, we found that TB neurons displayed distinct connections between the L‐AOTU columns and the BU groups (Figure 8m). Altogether, single TB neurons (Figure 8a–f) and specific TB drivers (Figure 2b6–b10 and Figure 9d1–d5) demonstrated the following wiring principles: (a) the BU_a_ receives visual inputs exclusively from the glomeruli in the L‐AOTU_3_ (Figure 8j); (b) the BU_s_ receive convergent visual inputs from the glomeruli in three consecutive columns (i.e., L‐AOTU_R2_ → BU_R‐s_, L‐AOTU_R3_ → BU_R‐s_, and L‐AOTU_R4_ → BU_R‐s_) (Figure 8h, k, and l); (c) the BU_i_ receives convergent visual inputs from the glomeruli in two consecutive columns (i.e., L‐AOTU_R1_ → BU_R‐i_ and L‐AOTU_R2_ → BU_R‐i_) (Figure 8g, i); and (d) each TB neuron has symmetrical neuronal partners in the contralateral brain hemisphere (i.e., L‐AOTU_R2‐d_ → BU_R‐s_ and L‐AOTU_L2‐d_ → BU_L‐s_) (Figure 11a, red line).

### 3.7 TT neurons

All TT neurons connected two L‐AOTUs between the two brain hemispheres (Figure 8n). Each TT neuron had dendritic arbors in the multiple glomeruli of two L‐AOTU middle columns and axon terminals in the same glomeruli of the contralateral L‐AOTU. Based on the differences in the connection between the L‐AOTU glomeruli of the two hemispheres, we classified TT neurons into two cell types belonging to one family in each hemisphere (Figure 6 and Tables 3 and 4). One cell type had dendrites innervating six glomeruli in two ipsilateral L‐AOTU columns, with the axons terminating in the symmetric glomeruli in two contralateral L‐AOTU columns (i.e., L‐AOTU_R2‐R3_ → L‐AOTU_L2‐L3_ or L‐AOTU_L2‐L3_ → L‐AOTU_R2‐R3_) (Figure 8n1). The other cell type had dendrites innervating three glomeruli in the ipsilateral L‐AOTU_3_, with the axons terminating in the symmetric glomeruli in the contralateral L‐AOTU_3_ (i.e., L‐AOTU_R3_ → L‐AOTU_L3_ or L‐AOTU_L3_ → L‐AOTU_R3_) (Figure 8n2). Altogether, single TT neurons (Figure 8o–p) and specific TT drivers (Figures 2c1–c2) showed the following wiring principle: two L‐AOTU middle columns receive and exchange the convergent visual inputs from MT neurons on both sides of the brain (Figure 11a, cyan line).
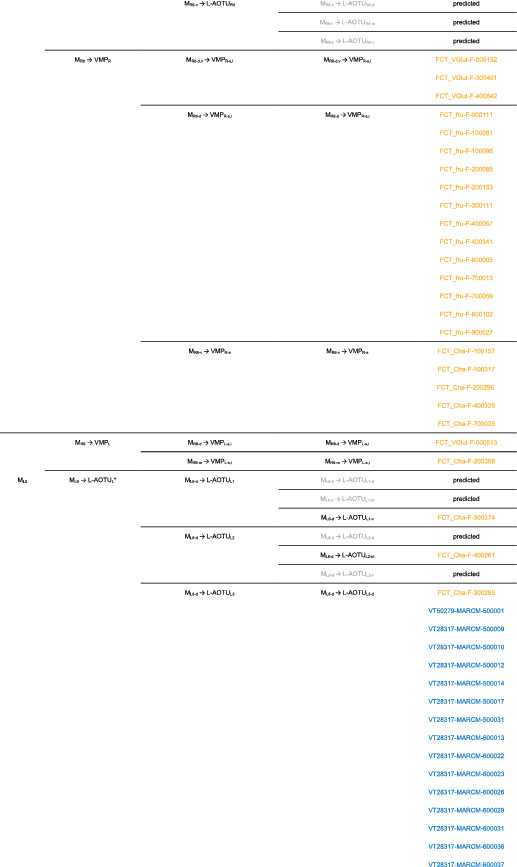
 
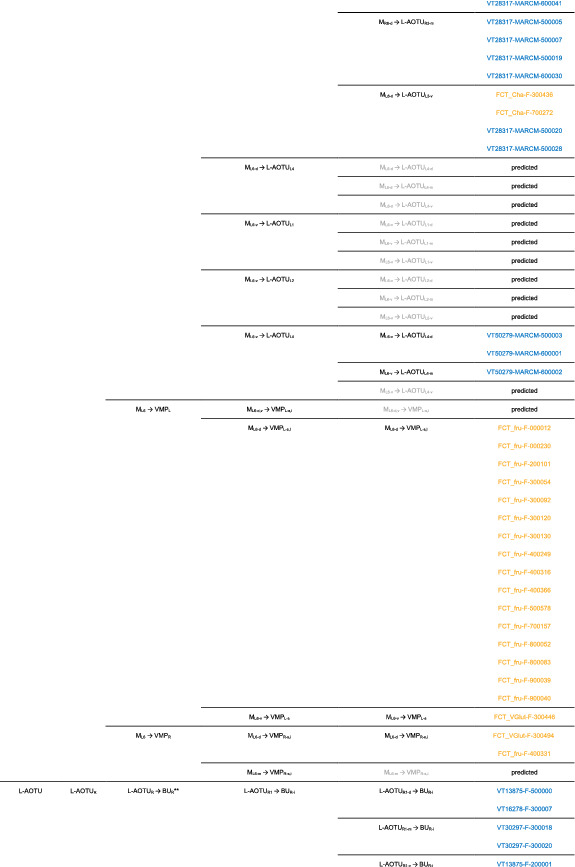
 
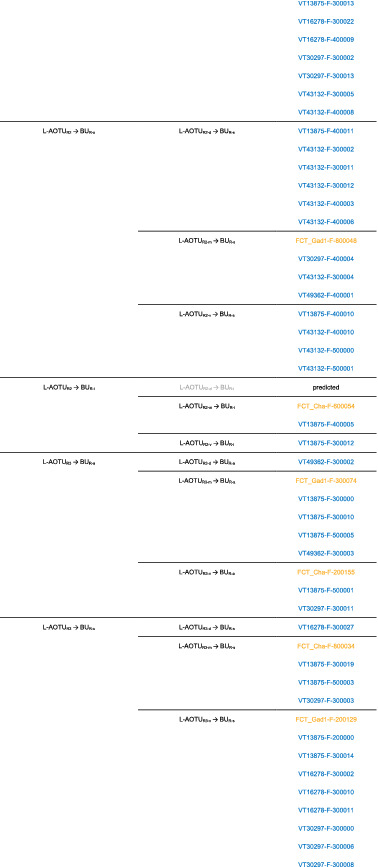
 
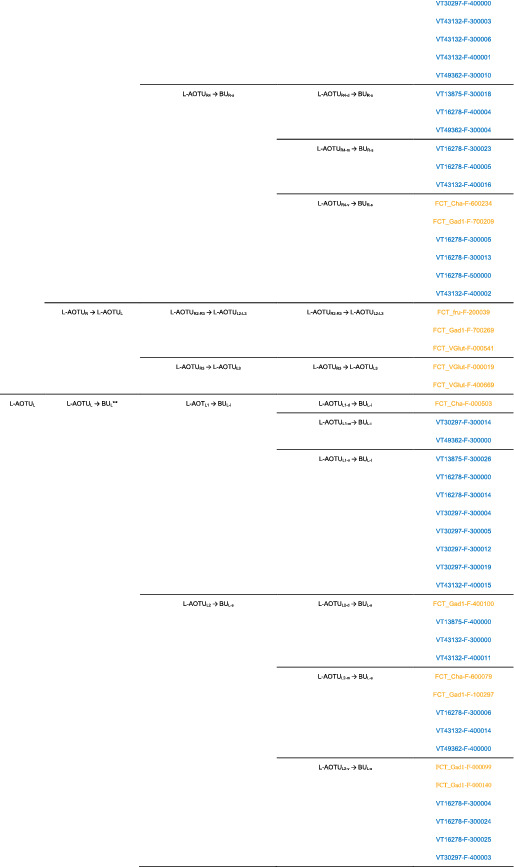
 
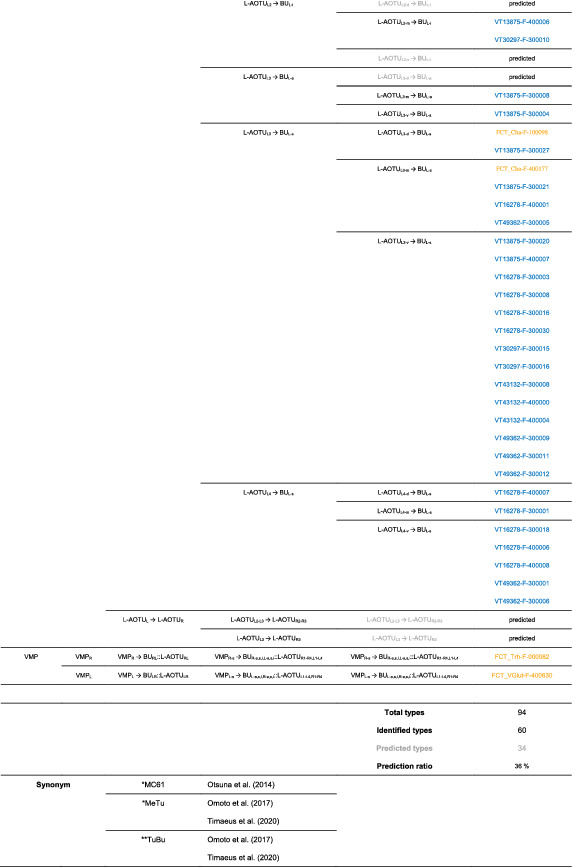
 
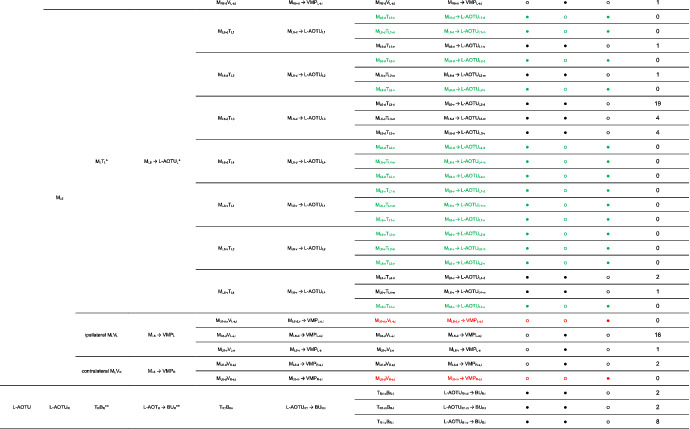
 
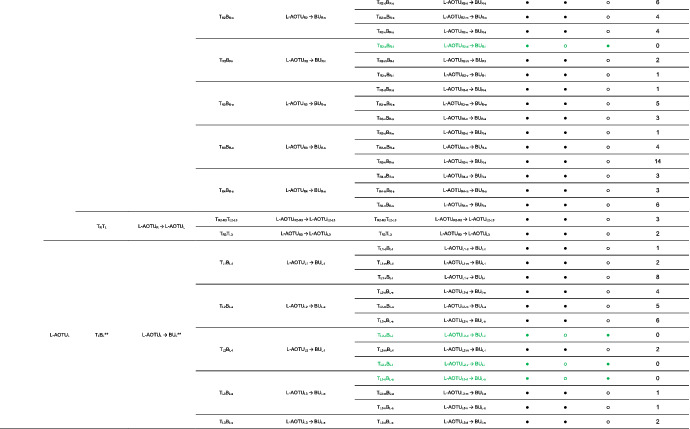
 
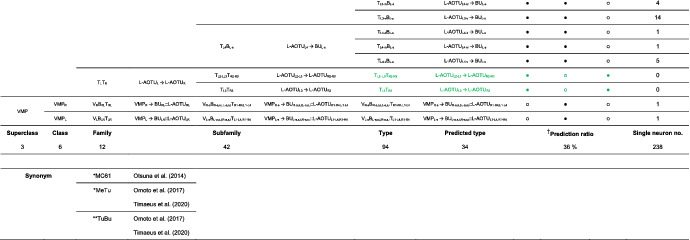


### 3.8 VBT neurons

**FIGURE 10:**
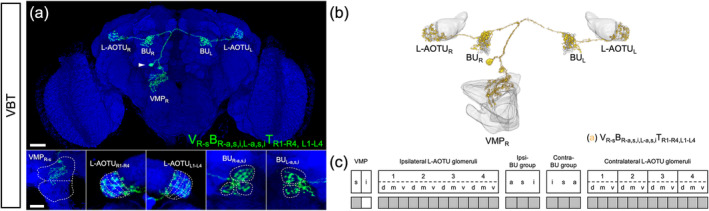
Connectivity analysis for ventral medial protocerebrum (VMP) output neurons VBT. (a) A representative VBT neuron (green). Brain structures immunostained with anti‐DLG antibodies (blue). (b) The VBT neuron is registered to a standard brain. (c) Gray indicates the innervation patterns of VBT neurons between VMP domains, L‐AOTU glomeruli, and BU groups. Images are frontal views of confocal projections of several adjacent optical sections unless otherwise specified. scale bars: 20 μm [Color figure can be viewed at wileyonlinelibrary.com]

**FIGURE 11:**
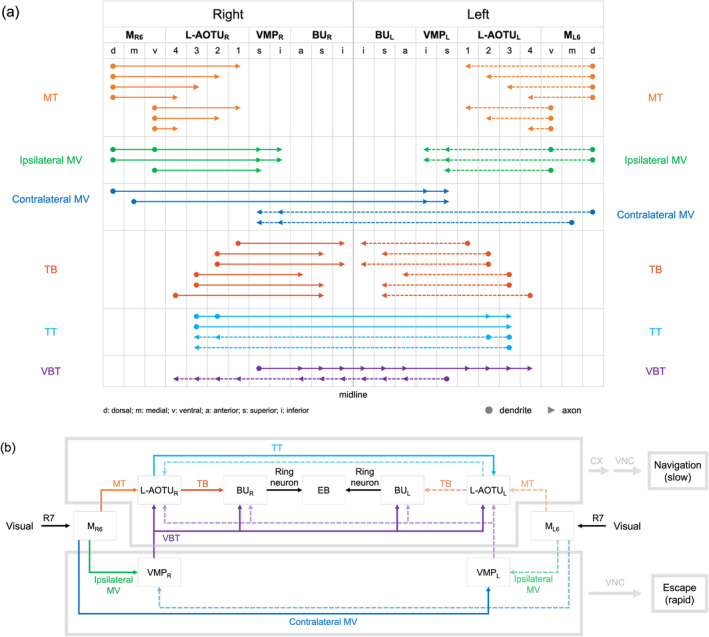
Summary of anatomical observations in UV pathways of the *Drosophila* brain. (a) Schematic overview displaying the projection organization in 12 families of visual projection neurons (VPNs). VPNs colored by family (MT neuron = yellow line, ipsilateral MV neuron = green line, contralateral MV = blue, TB neuron = red line, TT neuron = cyan line, and VBT neuron = purple line). VPNs on the right‐hand and the left‐hand side of the brain are shown in solid line and dotted line, respectively. Filled circles (dendritic) and arrowheads (axonal) point to the target regions of 42 subfamilies. (b) Wiring diagram of the UV circuitry. Arrows indicate the directions of visual information flow. Gray arrows indicate the directions of the purposed visual information flow. The purposed functional implications of the UV circuitry in the brain is shown in gray. M_6_, medulla layer 6; L‐AOTU, lateral anterior optic tubercle; VMP, ventral medial protocerebrum; BU, bulb; CX, central complex; VNC, ventral nerve cord; d, dorsal; m, medial; v, ventral; a, anterior; s, superior; i, inferior [Color figure can be viewed at wileyonlinelibrary.com]

The identification of the MV neurons allowed us to ask how visual information is conveyed to the EB from the VMP. Through the analysis of thousands of single neurons, we found an ascending neuron, the unique VMP output neuron, linking the unilateral VMP to the bilateral BU and the bilateral L‐AOTU (named VBT, Figure 10a). The VBT neurons had one cell type belonging to one subfamily in each hemisphere (Figure 6 and Tables 3 and 4). Each VBT neuron had dendritic arbors in the superior VMP and axon terminals in the three groups of the bilateral BU and further extended to four columns of the bilateral L‐AOTU (Figure 10b–c). Consequently, the VBT neuron had a symmetrical neuronal partner showing the mirror wiring diagram in the contralateral brain hemisphere (i.e., VMP_R‐s_ → BU_R‐a,s,i,L‐a,s,i_::L‐AOTU_R1‐R4,L1‐L4_ and VMP_L‐s_ → BU_L‐a,s,i,R‐a,s,i_::L‐AOTU_L1‐L4,R1‐R4_) (Figure 11a, purple line). These results seemed to indicate that each BU receives the visual inputs from the ipsilateral M_6_ and the contralateral M_6_ via three parallel pathways: a dorsal tract from the ipsilateral L‐AOTU and two medial tracts from the ipsilateral VMP and the contralateral VMP.

## 4 DISCUSSION

In this study, we provided a comprehensive map of single VPNs of the M_6_ downstream circuitry in the *Drosophila* brain. We identified and classified 238 VPNs into 94 cell types belonging to 12 families, based on their 3D morphology. Classification of these single neurons based on connectivity and specific Gal4 expression allowed us to refine distinct partitions of brain regions involved in UV information processing. The stereotyped circuit organization suggested that environment UV light is hierarchically represented in the brain, which integrates and processes UV information in a multilayer, differential, and bilateral manner.

**TABLE 6:** Lookup table of homologous visual projection neurons across species

Fruit fly			Locust, monarch butterfly, bumblebee, honeybee, silk moth	
Neuron name in the current study	Other name in the literatures	Reference	Homologous neurons in other species	Reference
**MT** (M_6_ → L‐AOTU)	**MeTu** (medullo‐tubercular neurons)	Omoto et al. (2017)	**TM** (transmedullary neurons)	**Locust**: Homberg et al. (2003)
		Timaeus et al. (2020)		**Butterfly**: Heinze et al. (2013)
	**MC61** (medullar columnar 61 neurons)	Otsuna et al. (2014)		**Bumblebee**: Pfeiffer and Kinoshita (2012)
	**TM** (transmedullary neurons)	Fischbach and Dittrich (1989)		**Honeybee**: Zeller et al. (2015)
**TB** (L‐AOTU → BU)	**TuBu** (tubercular‐bulbar neurons)	Omoto et al. (2017)	**TuLAL1** (tubercle‐accessory lobe 1 neurons)	**Locust**: Homberg et al. (2003)
		Timaeus et al. (2020)		**Butterfly**: Heinze et al. (2013)
		Lamaze et al. (2018)		**Bumblebee**: Pfeiffer and Kinoshita (2012)
		Guo et al. (2018)		**Honeybee**: Zeller et al. (2015)
**TT** (L‐AOTU → L‐AOTU)	**–**	**–**	**TuTu1** (tubercle‐tubercle neuron 1 neurons)	**Locust**: Homberg et al. (2003)
				**Butterfly**: Heinze et al. (2013)
				**Bumblebee**: Pfeiffer and Kinoshita (2012)
				**Honeybee**: Zeller et al. (2015)
**ipsilateral MV** (M_6_ → ipsi‐VMP)	**–**	**–**	Medulla output neurons (unnamed)	**Silk moth**: Namiki and Kanzaki (2018)

### 4.1 Homologous VPNs in other insect species

We morphologically identified six groups of visual projection neurons (VPNs) that connect the optic lobe to the central brain in *Drosophila* (Figure 1b–g). Several VPNs presented in this study have been previously described in various insect species, including locust (Homberg et al., 2003), butterfly (Heinze et al., 2013), bee (Pfeiffer & Kinoshita, 2012; Zeller et al., 2015), and silk moth (Namiki & Kanzaki, 2018). After performing comparative analysis of the neuronal morphology in the identified M_6_ downstream neurons, we provided a detailed list of the homologous VPNs in other insect species (Table 6). Three groups of VPNs are highly similar to the well‐characterized polarization‐sensitive neurons in locust (Homberg et al., 2003), butterfly (Heinze et al., 2013), and bees (Pfeiffer & Kinoshita, 2012; Zeller et al., 2015): (a) MT neurons are homologous to the transmedullary (TM) neurons with dendritic arbors in the medulla and axon terminals in the lower AOTU; (b) TB neurons are homologous to the tubercle‐accessory lobe 1 (TuLAL1) neurons with dendritic arbors in the lower AOTU and axon terminals in the lateral and medial bulb; and (c) TT neurons are homologous to the tubercle‐tubercle neuron 1 (TuTu1) neurons connecting the lower AOTUs of both hemispheres via the intertubercle tract. TT neurons in the fly are likely to be involved in polarization and color vision, as suggested by the functional studies of the TuTu1 neurons in the locust. Based on the size and location related to the surrounding neuropils, the VMP of the fly resembles the posterior slope of the silk moth. Thus, the ipsilateral MV neurons in *Drosophila* may also be involved in the mating behavior, as demonstrated in the morphologically similar medulla output neurons of the silk moth (Namiki & Kanzaki, 2018). With these anatomical observations, it can be stated that most of the visual projection neurons are highly conserved in different insect species (Kinoshita et al., 2007; Pfeiffer & Homberg, 2007).

**TABLE 7:** List of ellipsoid body (EB) ring neuron types

Neuron type	Innervation		Neurotransmitter	Reference
	EB rings^a^	BU groups^b^		
R1	C	i	GABA	Hanesch et al. (1989)
			Glutamate	Daniels et al. (2008)
			Acetylcholine	Zhang et al. (2013)
R2	A	s	GABA	Hanesch et al. (1989)
			Acetylcholine	Zhang et al. (2013)
R3	A	i	GABA	Hanesch et al. (1989)
			Glutamate	Daniels et al. (2008)
R4d	O	s	GABA	Hanesch et al. (1989)
R4m	O	a, s	GABA	Hanesch et al. (1989)
			Glutamate	RW Daniels et al. (2008)
			Acetylcholine	Zhang et al. (2013)
P	P	–	–	Lin et al. (2013)

^a^ Dendritic distribution in the specific EB ring is indicated: C, center ring; A, anterior ring; O, outer ring; P, posterior ring.

^b^ Axon distribution in the specific bulb (BU) groups is indicated: i, inferior groups; s, superior groups; a, anterior groups.

### 4.2 Internal representation of UV information at the L‐AOTU

Three independent lines of evidence showed that each L‐AOTU consisted of four columns, and every column consisted of three glomeruli: (a) evident anatomical boundary revealed by DLG immunostaining; (b) column‐specific Gal4 and LexA expression; and (c) single glomerulus‐specific input MT neurons and output TB neurons.

**FIGURE 12:**
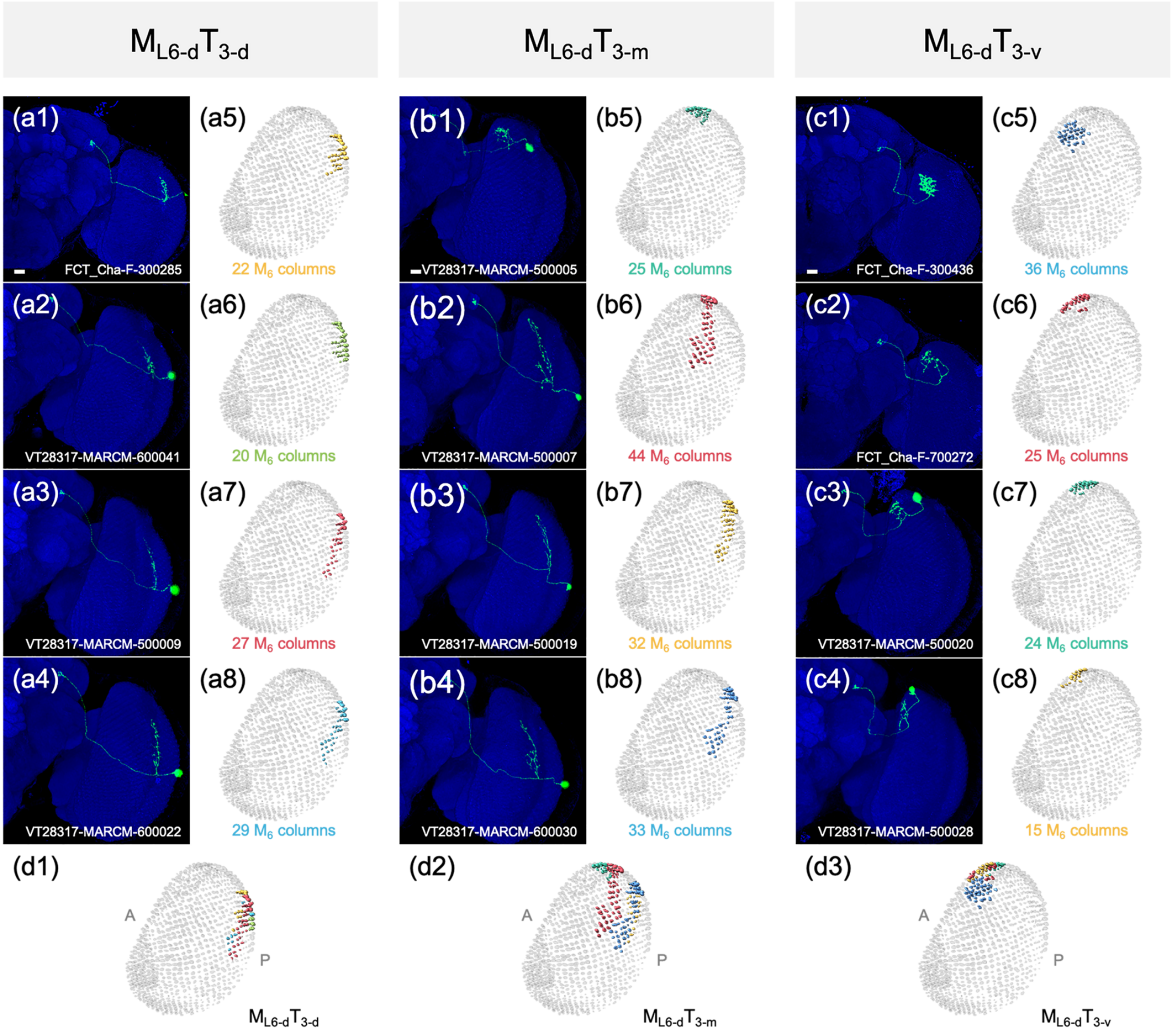
Retinotopic map of the M_L‐d_T_L3_ subfamily. (a) Four representative MT neurons of the M_L6‐d_T_3‐d_ type. Brain structures immunostained with anti‐DLG antibodies (blue). (b) Four representative MT neurons of the M_L6‐d_T_3‐m_ type. Brain structures immunostained with anti‐DLG antibodies (blue). (c) Four representative MT neurons of the M_L6‐d_T_3‐v_ type. Brain structures immunostained with anti‐DLG antibodies (blue). (a5–a8) Skeletal model showing the coverage of medulla layer 6 (M_6_) columns by individual M_L6‐d_T_3‐d_ types in a1–a4. (b5–b8) Skeletal model showing the coverage of M_6_ columns by individual M_L6‐d_T_3‐m_ types in b1–b4. (c5–c8) Skeletal model showing the coverage of M_6_ columns by individual M_L6‐d_T_3‐v_ types in c1–c4. (d) Merged skeletal model of the four M_L6‐d_T_3‐d_ (d1), four M_L6‐d_T_3‐m_ (d2), and four M_L6‐d_T_3‐v_ (d3). Images are frontal views of confocal projections of several adjacent optical sections unless otherwise specified. Scale bars: 20 μm [Color figure can be viewed at wileyonlinelibrary.com]

**FIGURE 13:**
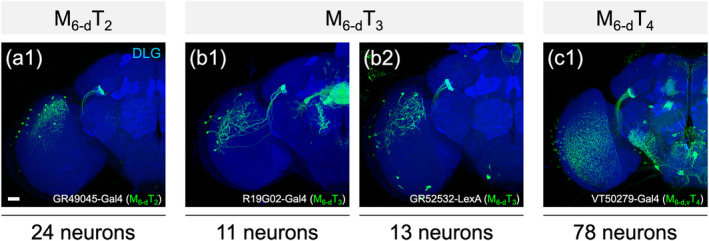
Number of column‐specific Gal4 and LexA drivers in three MT neurons in each brain hemisphere. Top: Neuron name of column‐specific MT neuron. Middle: Expression of column‐specific Gal4 and LexA drivers that were visualized with mCD8::GFP. Brain structures immunostained with anti‐DLG antibodies (blue). Bottom: Number of neurons for each column‐specific MT neuron by counting labeled cells in Gal4 and LexA drivers. Images are mostly frontal views of confocal projections of several adjacent optical sections unless otherwise specified. scale bars: 20 μm [Color figure can be viewed at wileyonlinelibrary.com]

As each UV‐sensing R7 neuron had an axonal bouton in a single M_6_ column, the terminals of all the R7 neurons of ~750 ommatidia form a retinotopic map at the M_6_ in a one‐to‐one pattern (Chin et al., 2014). The stereotypical connectivity of single MT neurons suggested the second layer of the internal representation of UV information to the 12 L‐AOTU glomeruli. As a single MT neuron had a dendritic arbor covering approximately 30 M_6_ columns and an axon terminal in one L‐AOTU glomerulus, an internal representation of UV information at the L‐AOTU required at least 25 MT neurons (Figure 12). However, a L‐AOTU single column‐specific driver was expressed in 10–80 MT neurons with dendritic arbors covering both dorsal and ventral M_6_, suggesting that each L‐AOTU column received UV information from the entire visual field (Figure 13). Thus, a L‐AOTU glomerulus may receive inputs from at least 8 MT neurons relaying UV information from approximately 250 M_6_ columns. Altogether, the M_6_ representation of the anterior–posterior visual field is likely further represented by the MT neurons along the dorsal–ventral axis of the three glomeruli in each L‐AOTU column. The calcium response of MT downstream neurons, that is, TB neurons and ring neurons of the EB, to horizontally moving objects suggested that each of the four L‐AOTU columns may separately be involved in processing azimuth of the visual field (Seelig & Jayaraman, 2013; Omoto et al., 2017).

The differential role of the four L‐AOTU columns remains unclear. Previous studies revealed three L‐AOTU columns: the medial L‐AOTU, the intermediate lateral L‐AOTU, and the lateral L‐AOTU (Omoto et al., 2017; Timaeus et al., 2020). With the single column‐specific driver expression and glomerulus‐specific single neuron morphology, we showed that the intermediate lateral L‐AOTU consists of two columns. Intriguingly, commissural TT neurons connected only the two middle columns between the two brain hemispheres. Similar commissural neurons were shown to be sensitive to polarized light, unpolarized light, and UV/green opponency in the locust brain (Kinoshita et al., 2007; Pfeiffer & Homberg, 2007). The distinct connectivity of individual TB neurons between single L‐AOTU glomeruli and single BU microglomeruli further supported the differential role of the four L‐AOTU columns. Moreover, functional imaging showed that the BU_s_ are sensitive to stimuli from the ipsilateral visual field and the BU_i_ to stimuli from the contralateral visual field (Omoto et al., 2017). Altogether, these results suggested that every L‐AOTU column receives inputs from the entire ipsilateral visual field. However, only the two middle L‐AOTU columns also receive inputs from the contralateral visual field, suggesting a differential role of the four L‐AOTU columns in the integration of UV information from the two eyes.

Furthermore, we predicted additional 34 types of M_6_ downstream neurons by examining thousands of single‐neuron images and driver expressions (See Table 4 for the list of predicted cell types in each family). These predicted neurons can be grouped into two groups comprised 32 and two cell types that were predicted from the specific drivers and the symmetrical neuronal partner, respectively. However, it should be noted that the neuronal types in each family might be incomplete, although we described a large number of visual projection neurons in this study. Neuronal types that connect L‐AOTU_1_ and L‐AOTU_3_ bilaterally across the two hemispheres may exist, as it has been shown that in honeybees, all compartments of the AOTUs of both hemispheres are connected via three types of TT neurons (Zeller et al., 2015). Our light microscopy data with electron microscopy reconstructions of the entire adult fruit fly brain could help to map the complete population of these visual projection neurons (Zheng et al., 2018).

### 4.3 Parallel processing and bilateral integration

Parallel circuit processing is a common feature of most sensory modalities, such as the visual and olfactory sensory systems. In *Drosophila*, the olfactory projection neurons relaying odor information to the mushroom body and the lateral horn were proposed to mediate learned and innate olfactory behaviors, respectively (Amin & Lin, 2019; Sanes & Zipursky, 2010). The M_6_ → L‐AOTU → EB is a common pathway processing polarized light for decision‐making during navigation in insects (el Jundi et al., 2014; Warren et al., 2019). Here, we found another pathway M_6_ → VMP with MV neurons relaying M_6_ information directly to the VMP premotor center, where dense descending neurons link the brain to the ventral nerve cord for rapid locomotion response (Namiki et al., 2018). In the silk moth *Bombyx mori*, head orientation requires a visual projection neuron with small‐field dendritic arbors in the medulla and large‐field axon terminals in the posterior slope, a brain region analogous to the VMP in the fly (Namiki & Kanzaki, 2018). The axon of MV neurons terminates in both the ipsilateral and the contralateral VMPs, where the VLT neurons further relay visual information to the BU and the L‐AOTU bilaterally, suggesting another circuit mechanism of bilateral integration of UV information from both eyes in the fruit fly. Whether the M_6_ → VMP → EB pathway mediates head orientation as in the silk moth remains to be addressed.

### 4.4 Heading representation

A population of EB output neurons (E‐PG) that is responsive to changes in head direction acts as an internal compass for navigation (Seelig & Jayaraman, 2015). The EB consists of five major types of intrinsic ring neurons (R1–R4 and P, Table 7). The R1–R4 neurons are downstream from TB neurons that connect at specific BU microglomeruli to the EB, while the P neurons connect the inferior dorsofrontal protocerebrum (IDFP) to the EB (Hanesch et al., 1989; Renn et al., 1999; Daniels et al., 2008; Lin et al., 2013; Zhang et al., 2013; Martín‐Peña et al., 2014). It remains unclear how EB ring neurons orchestrate the compass activity of EB output neurons. Here, we demonstrated parallel visual pathways and stereotyped connectivity from the M_6_ columns to the BU glomeruli, indicating a complex computation of bilateral UV information at the EB ring. To generate stable heading representation, GABAergic inhibition by the ring neurons on the E‐PG neurons is necessary (Fisher et al., 2019; Kim et al., 2019). However, while GABAergic R2 and R4m neurons convey information to the A ring and the O ring, respectively (Table 7), E‐PG neurons have dendrites only in the C ring and the P ring (Lin et al., 2013; Su et al., 2017). As every BU microglomerulus receives UV information, we speculated that heading representation is modulated by interactions among different EB ring neurons.

Parallel and bilateral visual pathways indicated that every EB ring receives visual inputs from bilateral BU glomeruli via ipsilateral pathways (MT–TB and MV–VBT) and contralateral pathways (MT–TT–TB and MV–VBT), suggesting different heading representations between the two BUs on each side of the EB ring. When a fly encounters a visual cue in the front, only the posterior M_6_ receives visual information from the R7 neurons in both eyes (Fischbach & Dittrich, 1989). From here, only dorsal glomeruli of the four L‐AOTU columns receive visual input from the posterior M_6_ in both brain hemispheres, indicating that some, but not all, BU microglomeruli receive visual inputs. As the visual cue moves to the right, both the posterior and the anterior M_6_ in the right brain hemisphere receive visual inputs. From here, all glomeruli in the four L‐AOTU columns in the right brain hemisphere receive visual inputs, indicating that all the microglomeruli of the right BU receive visual inputs from both the posterior and the anterior M_6_ in the right eye. The difference between the two M_6_ visual fields is further represented to the bilateral BUs by commissural TT neurons and ascending VBT neurons, suggesting a circuit mechanism for the heading representation during navigation.

Altogether, the morphological diversity of the visual projection neurons of the M_6_ downstream circuitry suggested multi‐dimensional processing of UV information via hierarchical, multiple‐layer, parallel, and bilateral circuits in the *Drosophila* brain. Notably, the downstream circuitry, which comprises the specialized polarization‐sensitive R7 neurons in the dorsal rim area (DRA) of the *Drosophila*'s eye terminate in the medulla dorsal rim area (MEDRA), may differ from the one described herein. Furthermore, the UV‐sensitive R7 and the blue‐green‐sensitive R8 neurons mutually inhibit each other (Schnaitmann et al., 2018). The M_6_ downstream circuitry may also be involved in color vision (with an antagonistic action) for green light, as has been shown for TT neurons in locusts (Kinoshita et al., 2007; Pfeiffer & Homberg, 2007). The UV‐sensitive R7 neurons also relay visual signals to the lobula via R7–Dm8–Tm5c for UV preference behavior (Gao et al., 2008; Karuppudurai et al., 2014), suggesting that the differentially processed downstream circuits of R7 neurons may convey diverse information to the central brain for color comparison in *Drosophila*. However, the mechanism underlying the circuit interactions between these parallel pathways (R7–Dm8–Tm5c, R7–MT–TB, and R7–MV–VBT) remains to be elucidated. Altogether, this comprehensive visual circuit map provides the basis for studying how the brain uses UV information to guide navigation behavior in *Drosophila*.

## PEER REVIEW

The peer review history for this article is available at https://publons.com/publon/10.1002/cne.25068.

## CONFLICT OF INTEREST

The authors declare no competing interests.

## AUTHOR CONTRIBUTIONS

Chu‐Yi Tai, An‐Lun Chin, and Ann‐Shyn Chiang planned the study and analyzed the data. Chu‐Yi Tai performed imaging experiments, *FlyCircuit* data analysis. Chu‐Yi Tai and Ann‐Shyn Chiang wrote the paper. Ann‐Shyn Chiang supervised the project. All authors had access to all the data in this study and take responsibility for the integrity of the data and the accuracy of the data analysis.

## Data Availability

The data that support the findings of this study are available from the corresponding author upon request.

## References

[cne25068-bib-0001] Amin, H. , & Lin, A. C. (2019). Neuronal mechanisms underlying innate and learned olfactory processing in *Drosophila* . Current Opinion in Insect Science, 36, 9–17. 10.1016/j.cois.2019.06.003. 31280185

[cne25068-bib-0002] Behnia, R. , & Desplan, C. (2015). Visual circuits in flies: Beginning to see the whole picture. Current Opinion in Neurobiology, 34, 125–132. 10.1016/j.conb.2015.03.010. 25881091 PMC4577302

[cne25068-bib-0003] Chiang, A. S. , Lin, C. Y. , Chuang, C. C. , Chang, H. M. , Hsieh, C. H. , Yeh, C. W. , Shih, C. T. , Wu, J. J. , Wang, G. T. , Chen, Y. C. , Wu, C. C. , Chen, G. Y. , Ching, Y. T. , Lee, P. C. , Lin, C. Y. , Lin, H. H. , Wu, C. C. , Hsu, H. W. , Huang, Y. A. , … Hwang, J. K. (2011). Three‐dimensional reconstruction of brain‐wide wiring networks in *Drosophila* at single‐cell resolution. Current Biology, 21, 1–11. 10.1016/j.cub.2010.11.056. 21129968

[cne25068-bib-0004] Chin, A. L. , Lin, C. Y. , Fu, T. F. , Dickson, B. J. , & Chiang, A. S. (2014). Diversity and wiring variability of visual local neurons in the *Drosophila* medulla M6 stratum. Journal of Comparative Neurology, 522, 3795–3816. 10.1002/cne.23622. PMC4265792 24782245

[cne25068-bib-0005] Daniels, R. W. , Gelfand, M. V. , Collins, C. A. , & DiAntonio, A. (2008). Visualizing glutamatergic cell bodies and synapses in *Drosophila* larval and adult CNS. Journal of Comparative Neurology, 508, 131–152. 10.1002/cne.21670. 18302156

[cne25068-bib-0006] el Jundi, B. , Pfeiffer, K. , Heinze, S. , & Homberg, U. (2014). Integration of polarization and chromatic cues in the insect sky compass. Journal of Comparative Physiology A, 200, 575–589. 10.1007/s00359-014-0890-6. 24589854

[cne25068-bib-0007] Feinberg, E. H. , VanHoven, M. K. , Bendesky, A. , Wang, G. , Fetter, R. D. , Shen, K. , & Bargmann, C. I. (2008). GFP reconstitution across synaptic partners (GRASP) defines cell contacts and synapses in living nervous systems. Neuron, 57, 353–363. 10.1016/j.neuron.2007.11.030. 18255029

[cne25068-bib-0008] Fischbach, K. F. , & Dittrich, A. P. M. (1989). The optic lobe of *Drosophila melanogaster*. I. a Golgi analysis of wild‐type structure. Cell and Tissue Research, 258, 441–475. 10.1007/BF00218858.

[cne25068-bib-0009] Fisher, Y. E. , Lu, J. , D'Alessandro, I. , & Wilson, R. I. (2019). Sensorimotor experience remaps visual input to a heading‐direction network. Nature, 576, 121–125. 10.1038/s41586-019-1772-4. 31748749 PMC7753972

[cne25068-bib-0010] Gao, S. , Takemura, S. , Ting, C. Y. , Huang, S. , Lu, Z. , Luan, H. , Rister, J. , Thum, A. S. , Yang, M. , Hong, S. T. , Wang, J. W. , Odenwald, W. F. , White, B. H. , Meinertzhagen, I. A. , & Lee, C. H. (2008). The neural substrate of spectral preference in *Drosophila* . Neuron, 60, 328–342. 10.1016/j.neuron.2008.08.010. 18957224 PMC2665173

[cne25068-bib-0040] Guo, F., Holla, M., Díaz, M. M., & Rosbash, M. (2018). A circadian output circuit controls sleep‐wake arousal in *Drosophila*. Neuron, 100, 624–635. 10.1016/j.neuron.2018.09.002 30269992

[cne25068-bib-0011] Hanesch, U. , Fischbach, K. F. , & Heisenberg, M. (1989). Neuronal architecture of the central complex in *Drosophila melanogaster* . Cell and Tissue Research, 257, 343–366. 10.1007/BF00261838.

[cne25068-bib-0012] Heinze, S. , Florman, J. , Asokaraj, S. , el Jundi, B. , & Reppert, S. M. (2013). Anatomical basis of sun compass navigation II: The neuronal composition of the central complex of the monarch butterfly. Journal of Comparative Neurology, 521, 267–298. 10.1002/cne.23214. 22886450

[cne25068-bib-0013] Homberg, U. , Hofer, S. , Pfeiffer, K. , & Gebhardt, S. (2003). Organization and neural connections of the anterior optic tubercle in the brain of the locust, *Schistocerca gregaria* . Journal of Comparative Neurology, 462, 415–430. 10.1002/cne.10771. 12811810

[cne25068-bib-0014] Karuppudurai, T. , Lin, T. Y. , Ting, C. Y. , Pursley, R. , Melnattur, K. V. , Diao, F. , White, B. H. , Macpherson, L. J. , Gallio, M. , Pohida, T. , & Lee, C. H. (2014). A hard‐wired glutamatergic circuit pools and relays UV signals to mediate spectral preference in *Drosophila* . Neuron, 81, 603–615. 10.1016/j.neuron.2013.12.010. 24507194 PMC3920195

[cne25068-bib-0015] Kim, S. S. , Hermundstad, A. M. , Romani, S. , Abbott, L. F. , & Jayaraman, V. (2019). Generation of stable heading representations in diverse visual scenes. Nature, 576, 126–131. 10.1038/s41586-019-1767-1. 31748750 PMC8115876

[cne25068-bib-0016] Kinoshita, M. , Pfeiffer, K. , & Homberg, U. (2007). Spectral properties of identified polarized‐light sensitive interneurons in the brain of the desert locust *Schistocerca gregaria* . Journal of Experimental Biology, 210, 1350–1361. 10.1242/jeb.02744. 17401118

[cne25068-bib-0041] Lamaze, A., Krätschmer, P., Chen, K. F., Lowe, S., & Jepson, J. E. (2018). A wake‐promoting circadian output circuit in *Drosophila*. Current Biology, 28, 3098–3105. 10.1016/j.cub.2018.07.024 30270186

[cne25068-bib-0017] Lee, T. , & Luo, L. (1999). Mosaic analysis with a repressible cell marker for studies of gene function in neuronal morphogenesis. Neuron, 22, 451–461. 10.1016/S0896-6273(00)80701-1. 10197526

[cne25068-bib-0018] Lin, C. Y. , Chuang, C. C. , Hua, T. E. , Chen, C. C. , Dickson, B. J. , Greenspan, R. J. , & Chiang, A. S. (2013). A comprehensive wiring diagram of the protocerebral bridge for visual information processing in the *Drosophila* brain. Cell Reports, 3, 1739–1753. 10.1016/j.celrep.2013.04.022. 23707064

[cne25068-bib-0019] Martín‐Peña, A. , Acebes, A. , Rodríguez, J. R. , Chevalier, V. , Casas‐Tinto, S. , Triphan, T. , Strauss, R. , & Ferrús, A. (2014). Cell types and coincident synapses in the ellipsoid body of *Drosophila* . European Journal of Neuroscience, 39, 1586–1601. 10.1111/ejn.12537. 24605774

[cne25068-bib-0020] Namiki, S. , Dickinson, M. H. , Wong, A. M. , Korff, W. , & Card, G. M. (2018). The functional organization of descending sensory‐motor pathways in *Drosophila* . eLife, 7, e34272. 10.7554/eLife.34272. 29943730 PMC6019073

[cne25068-bib-0021] Namiki, S. , & Kanzaki, R. (2018). Morphology of visual projection neurons supplying premotor area in the brain of the silkmoth *Bombyx mori* . Cell and Tissue Research, 374, 497–515. 10.1007/s00441-018-2892-0. 30078100

[cne25068-bib-0022] Omoto, J. J. , Keleş, M. F. , Nguyen, B. C. M. , Bolanos, C. , Lovick, J. K. , Frye, M. A. , & Hartenstein, V. (2017). Visual input to the *Drosophila* central complex by developmentally and functionally distinct neuronal populations. Current Biology, 27, 1098–1110. 10.1016/j.cub.2017.02.063. 28366740 PMC5446208

[cne25068-bib-0023] Otsuna, H. , Shinomiya, K. , & Ito, K. (2014). Parallel neural pathways in higher visual centers of the *Drosophila* brain that mediate wavelength‐specific behavior. Frontiers in Neural Circuits, 8, 8. 10.3389/fncir.2014.00008. 24574974 PMC3918591

[cne25068-bib-0024] Pfeiffer, K. , & Homberg, U. (2007). Coding of azimuthal directions via time‐compensated combination of celestial compass cues. Current Biology, 17, 960–965. 10.1016/j.cub.2007.04.059. 17524646

[cne25068-bib-0025] Pfeiffer, K. , & Kinoshita, M. (2012). Segregation of visual inputs from different regions of the compound eye in two parallel pathways through the anterior optic tubercle of the bumblebee (*Bombus ignitus*). Journal of Comparative Neurology, 520, 212–229. 10.1002/cne.22776. 21953619

[cne25068-bib-0026] Renn, S. C. P. , Armstrong, J. D. , Yang, M. , Wang, Z. , An, X. , Kaiser, K. , & Taghert, P. H. (1999). Genetic analysis of the *Drosophila* ellipsoid body neuropil: Organization and development of the central complex. Journal of Neurobiology, 41, 189–207. 10.1002/(SICI)1097-4695(19991105)41:2<189::AID-NEU3>3.0.CO;2-Q. 10512977

[cne25068-bib-0027] Sanes, J. R. , & Zipursky, S. L. (2010). Design principles of insect and vertebrate visual systems. Neuron, 66, 15–36. 10.1016/j.neuron.2010.01.018. 20399726 PMC2871012

[cne25068-bib-0028] Schnaitmann, C. , Haikala, V. , Abraham, E. , Oberhauser, V. , Thestrup, T. , Griesbeck, O. , & Reiff, D. F. (2018). Color processing in the early visual system of *Drosophila* . Cell, 172, 318–330. 10.1016/j.cell.2017.12.018. 29328919

[cne25068-bib-0029] Seelig, J. D. , & Jayaraman, V. (2013). Feature detection and orientation tuning in the *Drosophila* central complex. Nature, 503, 262–266. 10.1038/nature12601. 24107996 PMC3830704

[cne25068-bib-0030] Seelig, J. D. , & Jayaraman, V. (2015). Neural dynamics for landmark orientation and angular path integration. Nature, 521, 186–191. 10.1038/nature14446. 25971509 PMC4704792

[cne25068-bib-0031] Shih, C. T. , Sporns, O. , Yuan, S. L. , Su, T. S. , Lin, Y. J. , Chuang, C. C. , Wang, T. Y. , Lo, C. C. , Greenspan, R. J. , & Chiang, A. S. (2015). Connectomics‐based analysis of information flow in the *Drosophila* brain. Current Biology, 25, 1249–1258. 10.1016/j.cub.2015.03.021. 25866397

[cne25068-bib-0032] Song, B. M. , & Lee, C. H. (2018). Toward a mechanistic understanding of color vision in insects. Frontiers in Neural Circuits, 12, 16. 10.3389/fncir.2018.00016. 29527156 PMC5829095

[cne25068-bib-0033] Su, T. S. , Lee, W. J. , Huang, Y. C. , Wang, C. T. , & Lo, C. C. (2017). Coupled symmetric and asymmetric circuits underlying spatial orientation in fruit flies. Nature Communications, 8, 1–15. 10.1038/s41467-017-00191-6. PMC5529380 28747622

[cne25068-bib-0034] Takemura, S. , Bharioke, A. , Lu, Z. , Nern, A. , Vitaladevuni, S. , Rivlin, P. K. , Katz, W. T. , Olbris, D. J. , Plaza, S. M. , Winston, P. , Zhao, T. , Horne, J. A. , Fetter, R. D. , Takemura, S. , Blazek, K. , Chang, L. A. , Ogundeyi, O. , Saunders, M. A. , Shapiro, V. , … Chklovskii, D. B. (2013). A visual motion detection circuit suggested by *Drosophila* connectomics. Nature, 500, 175–181. 10.1038/nature12450. 23925240 PMC3799980

[cne25068-bib-0035] Timaeus, L. , Geid, L. , Sancer, G. , Wernet, M. F. , & Hummel, T. (2020). Parallel visual pathways with topographic versus non‐topographic organization connect the *Drosophila* eyes to the central brain. *bioRxiv*, 2020.04.11.037333. 10.1101/2020.04.11.037333 PMC7648135 33205011

[cne25068-bib-0036] Warren, T. L. , Giraldo, Y. M. , & Dickinson, M. H. (2019). Celestial navigation in *Drosophila* . Journal of Experimental Biology, 222. 10.1242/jeb.186148. PMC7375828 30728228

[cne25068-bib-0037] Zeller, M. , Held, M. , Bender, J. , Berz, A. , Heinloth, T. , Hellfritz, T. , & Pfeiffer, K. (2015). Transmedulla neurons in the sky compass network of the honeybee (*Apis mellifera*) are a possible site of circadian input. PLoS One, 10, e0143244. 10.1371/journal.pone.0143244. 26630286 PMC4667876

[cne25068-bib-0038] Zhang, Z. , Li, X. , Guo, J. , Li, Y. , & Guo, A. (2013). Two clusters of GABAergic ellipsoid body neurons modulate olfactory labile memory in *Drosophila* . Journal of Neuroscience, 33, 5175–5181. 10.1523/JNEUROSCI.5365-12.2013. 23516283 PMC6705003

[cne25068-bib-0039] Zheng, Z. , Lauritzen, J. S. , Perlman, E. , Robinson, C. G. , Nichols, M. , Milkie, D. , Torrens, O. , Price, J. , Fisher, C. B. , Sharifi, N. , Calle‐Schuler, S. A. , Kmecova, L. , Ali, I. J. , Karsh, B. , Trautman, E. T. , Bogovic, J. A. , Hanslovsky, P. , Jefferis, G. S. X. E. , Kazhdan, M. , … Bock, D. D. (2018). A complete electron microscopy volume of the brain of adult *Drosophila melanogaster* . Cell, 174, 730–743. 10.1016/j.cell.2018.06.019. 30033368 PMC6063995

